# Metabolomics for early detection of stress in freshwater alga *Poterioochromonas malhamensis* exposed to silver nanoparticles

**DOI:** 10.1038/s41598-020-77521-0

**Published:** 2020-11-25

**Authors:** Wei Liu, Sanghamitra Majumdar, Weiwei Li, Arturo A. Keller, Vera I. Slaveykova

**Affiliations:** 1 Department F.-A. Forel for Environmental and Aquatic Sciences, Environmental Biogeochemistry and Ecotoxicology, Faculty of Sciences, Earth and Environment Sciences, University of Geneva, Uni Carl Vogt,66 Blvd Carl-Vogt, 1211 Geneva, Switzerland; 2 Bren School of Environmental Science and Management, University of California, Santa Barbara, CA 93106-5131 USA

**Keywords:** Environmental impact, Metabolomics

## Abstract

Silver nanoparticles (AgNPs) are one of the most used engineered nanomaterials. Despite progress in assessing their environmental implications, knowledge gaps exist concerning the metabolic perturbations induced by AgNPs on phytoplankton, essential organisms in global biogeochemical cycles and food-web dynamics. We combine targeted metabolomics, biouptake and physiological response studies to elucidate metabolic perturbations in alga *Poterioochromonas malhamensis* induced by AgNPs and dissolved Ag. We show time-dependent perturbation of the metabolism of amino acids, nucleotides, fatty acids, tricarboxylic acids, photosynthesis and photorespiration by both Ag-treatments. The results suggest that dissolved Ag ions released by AgNPs are the major toxicity driver; however, AgNPs internalized in food vacuoles contributed to the perturbation of amino acid metabolism, TCA cycle and oxidative stress. The metabolic perturbations corroborate the observed physiological responses. We highlight the potential of metabolomics as a tool for understanding the molecular basis for these metabolic and physiological changes, and for early detection of stress.

## Introduction

Metabolomics tracks the changes in low-molecular-weight metabolites involved in different biological reactions^1,2^ under different environmental stimuli and stressors. It is well suited to study organism–environment interactions^3^ and serves to integrate information on metabolic pathway perturbation with physiological responses to stressors. Nanoparticle-induced metabolic perturbations to environmentally relevant organisms, such as plants, have only been recently studied systematically by both untargeted and targeted metabolomics^4–9^. The perspectives and challenges in the application of metabolomics to understand the nanotoxicology of plants have recently been comprehensively reviewed^10,11^. Regarding phytoplankton, only one metabolomic study focused on the effect of silver nanoparticles (AgNPs) on cyanobacterium *Microcystis aeruginosa*^12^. Hence, there is a significant knowledge gap with respect to the metabolic perturbations induced by engineered nanomaterials on phytoplankton, despite their pivotal role in global biogeochemical cycles and food-web dynamics^13^.

The present study focusses on AgNPs as they are one of the most extensively used materials in a variety of consumer products, mainly due to their biocidal properties^14^. The release of AgNPs from different products^15^ has raised significant concerns about possible consequences to the aquatic environment^16^. Indeed, the potential of AgNPs to affect phytoplankton community structure and functioning^17,18^, as well as individual phytoplankton species^19,20^ is well documented. Dissolution, uptake, oxidative stress^21,22^ and photosynthesis inhibition^17,19,23^ were found as the major drivers of AgNP toxicity. However, the extent of uptake and dissolution of AgNPs, and their contribution to the overall toxicity in phytoplankton community remains unclear. For example, released Ag ions rather than AgNPs were responsible for the toxicity to green algae *Raphidocelis subcapitata*^24^ and *Chlamydomonas reinhardtii*^25^; however, AgNPs directly contributed to the toxicity in chrysophyte *Ochromonas dania*^26^. AgNPs were also found in the cytoplasm of *C. reinhardtii*^27^, but the possible alterations of algal metabolism were not explored. While it is useful to study the physiological responses of AgNPs, there is a need to understand how AgNPs affect the metabolic processes of these organisms. Hence, metabolomics can serve to reveal these subtle but very important effects.

The primary goal of the current study is to obtain new insights into metabolic perturbations underlying the cellular responses in algae exposed to AgNPs. Using targeted metabolomics we address the following research questions: (1) what are the major metabolic pathways of algae that are influenced by AgNPs?; (2) what is the time progression of the metabolic perturbations?; (3) are they common or different for AgNPs and dissolved Ag treatments?

We examine the interactions of citrate-coated 20 nm AgNPs with the freshwater alga *Poterioochromonas malhamensis*, as representative of freshwater phytoplankton. This golden-brown alga often dominates mixotrophic phytoplankton populations^28^. *P. malhamensis* uses a wide selection of different organic food sources via phagotrophy or osmotrophy^29^. Hence, we assume this alga can take up AgNPs and their aggregates via phagocytosis. It also plays an important role in the fate and transport of organic matter in the environment^30,31^. Citrate-coated AgNPs were chosen in the present study according to the recommendations of the Organization for Economic Cooperation and Development^32^. Liquid chromatography–mass spectrometry (LC–MS)-based targeted metabolomics was used to quantify selected primary and secondary metabolites and their modulation by AgNPs and dissolved Ag treatments. In parallel, physiological responses such as enhanced generation of reactive oxygen species (ROS), lipid peroxidation and changes in photosynthetic activity were assessed. Uptake of AgNPs by *P. malhamensis*, together with key characteristics of AgNPs in the exposure medium were determined. The results highlight the high added value of metabolomics to elucidate the mechanisms that cause the physiological response in phytoplankton induced by stressors such as engineered nanomaterials.

## Results and discussion

### Characterization of AgNPs suspensions in the exposure medium

**Figure 1:**
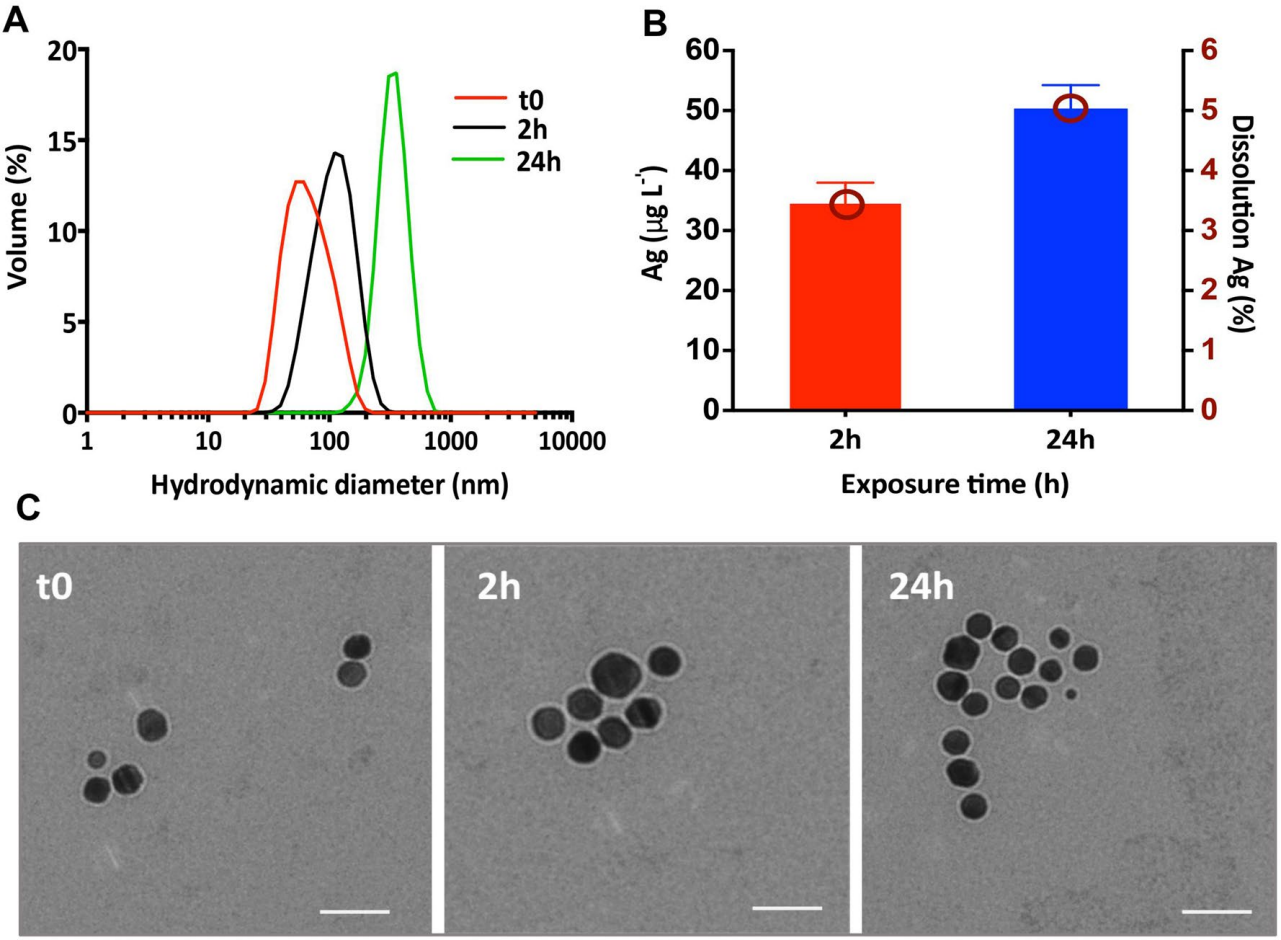
Characterization of the suspensions of AgNPs in the exposure medium at t0, 2 h and 24 h. (**A**) AgNPs hydrodynamic size distribution obtained by DLS as volume %; (**B**) AgNPs dissolution in Waris-H medium. Concentration and percentage of dissolved Ag in 1 mg L^−1^ AgNP suspensions; (**C**) TEM images of AgNPs in the exposure medium at t0, 2 h and 24 h. Scale bars: 50 nm.

The suspensions of AgNPs were characterized in terms of hydrodynamic size distribution and average diameter, zeta potential (ZP) and dissolution. Dispersion of 1 mg L^−1^ AgNPs in the exposure medium, resulted in an immediate formation of aggregates with a peak of the hydrodynamic size distribution centered at 61 ± 5 nm, and ZP of − 23 ± 3 mV. The size distributions shifted towards higher values with a maximum at 127 ± 6 nm (ZP: − 20 ± 2 mV) at 2 h and 350 ± 3 nm (ZP: − 12 ± 2 mV) at 24 h (Fig. 1A). These results indicate that the AgNPs aggregated in the exposure medium with time. The above results are consistent with transmission electron microscopy (TEM) observations showing spherical AgNPs with a size of 20 nm and aggregated forms (Fig. 1C).

Dissolution of the AgNPs was low with dissolved Ag representing ~ 2.50% and 4.07% of the total Ag in the suspensions after 2 h and 24 h, respectively (Fig. 1B). These values were comparable with previously reported results in other exposure media specific to algal bioassays^25,26,33–35^.

### Ag cellular burden and internalization of AgNPs by *P. malhamensis*

**Figure 2:**
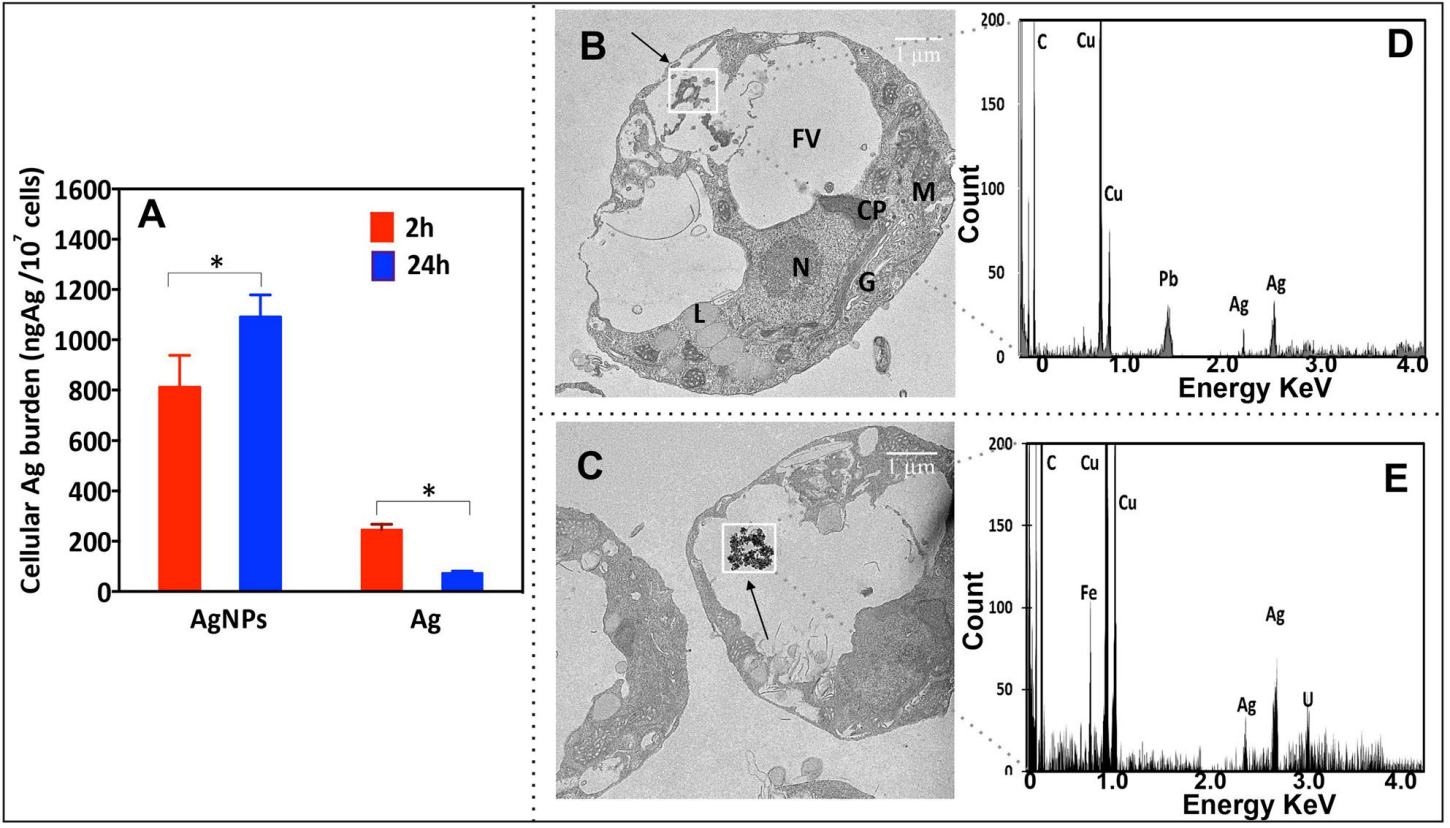
(**A**) Ag cellular burden of *P. malhamensis* exposed to 1.0 mg L^−1^ AgNPs or 40.7 μg L^−1^ AgNO_3_ during 2 h and 24 h. The results are presented as ng of cellular Ag per 1 × 10^7^ cells. Asterisk indicates a significant difference between treatments obtained by two-way analysis of variance (ANOVA) followed by a Sidak’s multiple comparisons test (*p* < 0.05); (**B**,**C**) TEM images of *P. malhamensis*: treated with 1 mg L^−1^ AgNPs for 2 h (**B**) and for 24 h (**C**); (**D**,**E**) energy dispersive X-ray (EDX) spectra correspond to the interest area from C (**D**) and D (**E**). Letters on (**B**) denote cell organelles: *G* Golgi body, *M* mitochondrion, *N* nucleus, *FV* food vacuole, *L* lipid drops, *CP* chloroplast. *p < 0.05, **p < 0.01, ***p < 0.001.

To quantify cellular Ag cellular burden and explore the internalization of AgNPs, we combined ICP-MS analysis of the algal cells for total cellular Ag concentrations and 2D TEM of the ultrathin cell sections. Exposure to AgNPs led to a significant accumulation of Ag in the algal cells, which increased with exposure time (Fig. 2A). Silver cellular burden was higher in AgNP treatments compared with exposures to AgNO_3_, and increased with exposure duration from 2 to 24 h. The contribution of the dissolved Ag present in the suspensions of AgNPs to the overall Ag was low (Fig. 2A). Ag cellular burden of *P. malhamensis* exposed to dissolved Ag at 24 h was lower than at 2 h, which could be related to decrease in the exposure medium, following significant cellular accumulation, as well as “growth-dilution effect” a kinetic effect in which accumulated metals are diluted within the algal cell by photosynthetically fixed carbon^36^.

TEM revealed the presence of aggregates with size around 100 nm in the food vacuoles of *P. malhamensis* exposed to AgNPs for 2 h (Fig. 2B). The above observation was confirmed in the 24 h exposure, where even larger aggregates of around 500 nm were found in the vacuoles (Fig. 2C). The characteristic peak of Ag L_α1_ and L_β1_ observed in EDX spectra confirmed that the aggregates contained Ag (Fig. 2D,E). The present results revealed that *P. malhamensis* internalized AgNPs, probably by phagocytosis. Indeed *P. malhamensis* is known to ingest plankton by phagocytosis^37^. Hence, the algae could possibly misidentify AgNPs and their aggregates as nutritive particulate organic matter and thus ingest them in a similar way. The above results are consistent with the observation that AgNPs are internalized in the cells of another species, *Ochromonas danica*^26^, which has been previously shown to have endocytosis^31^. However, it remains unclear whether AgNPs inside the cells alter algal metabolism directly or indirectly by the release of Ag ions.

### Effect of Ag-treatments on algal physiology

**Figure 3:**
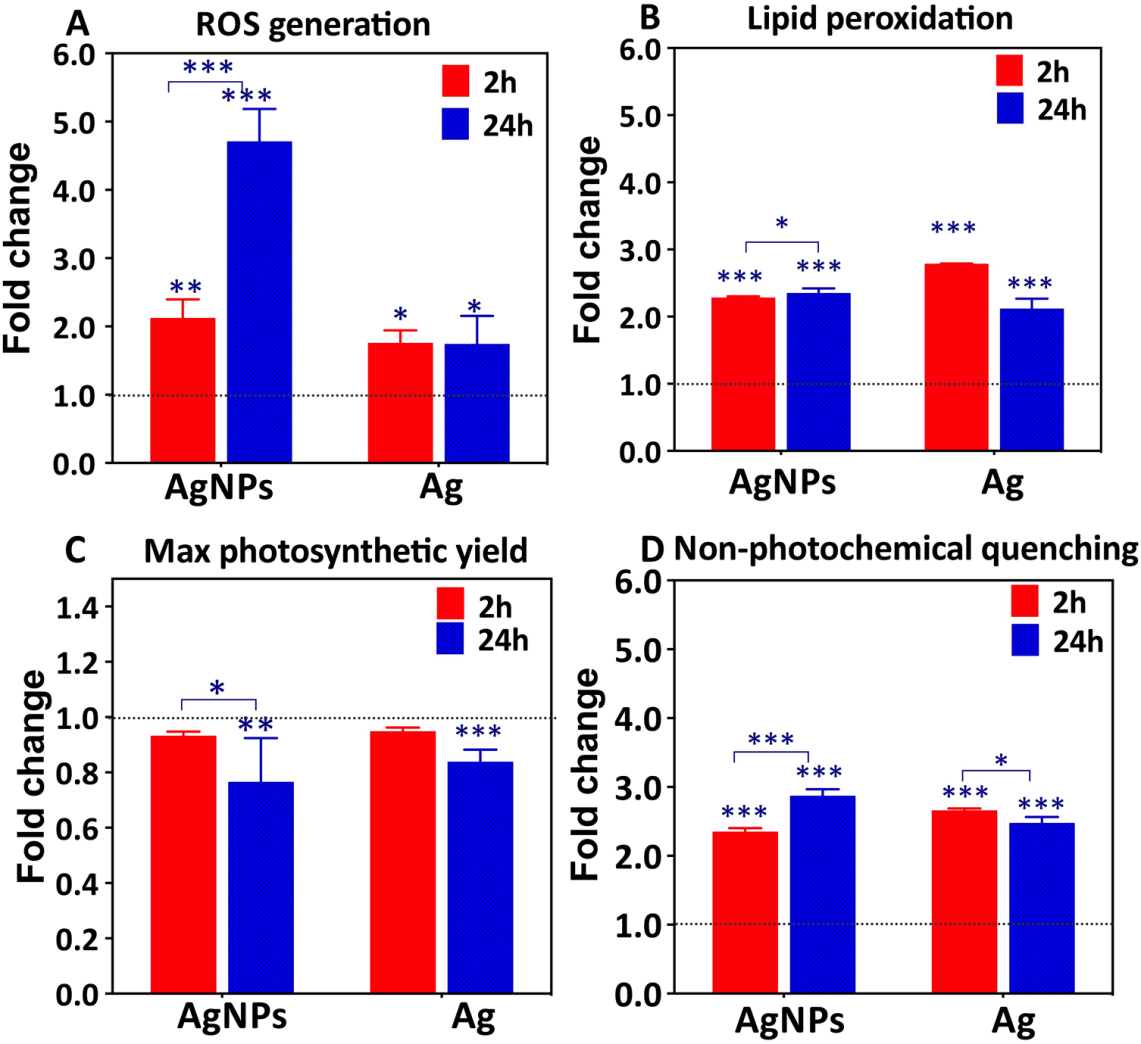
Effect of 1.0 mg L^−1^ AgNPs or 40.7 μg L^−1^ AgNO_3_ exposure on physiology of *P. malhamensis*. (**A**) ROS generation, determined by CellRoxGreen stain and FCM; (**B**) lipid peroxidation, assessed by MDA test; (**C**) maximum quantum yield of photosystem II (Fv/Fm); and (**D**) non-photochemical quenching (NPQ). Fold change is calculated as a ratio of the respective effects observed in Ag—treated cells and untreated control. Asterisks indicate a significant difference between treatments obtained by two-way analysis of variance (ANOVA) followed by a Sidak’s multiple comparisons test: *p < 0.05; ***p < 0.01; ***p < 0.001.

Exposure to AgNPs resulted in a significant and time-dependent increase of cellular ROS generation. The percentage of cells with enhanced ROS production increased to 2.1 ± 0.3 at 2 h and 4.7 ± 0.5 times at 24 h exposure, in comparison with unexposed controls (Fig. 3A). AgNP concentration-dependent increase of the percentage of cells with excessive ROS was also evidenced (see supplementary information). These results are consistent with the enhanced ROS generation observed in *Chlorella vulgaris* and *Dunaliella tertiolecta* exposed to AgNPs^38^. Exposure to AgNO_3_ at a concentration corresponding to the dissolved Ag in the AgNP suspensions resulted in about 1.8 ± 0.2 time increase of cells with enhanced ROS, which was time independent. The results also revealed that AgNPs, rather than dissolved Ag present in the AgNP suspensions, contributed significantly to cellular ROS generation, particularly at 24 h exposure (Fig. 3A). Excessive levels of ROS could lead to cellular oxidative stress and damage such as lipid peroxidation^22^. Indeed, both Ag-treatments induced lipid peroxidation in *P. malhamensis* (Fig. 3B).

Exposure to AgNPs resulted in about 2.27 ± 0.03 times increase of lipid peroxidation at 2 h as compared with unexposed controls, which was unchanged at 24 h exposure (2.34 ± 0.07 times increase). Exposure to dissolved Ag resulted in 2.78 ± 0.10 times increase in the cells with lipid peroxidation at 2 h and 2.11 ± 0.15 times at 24 h, as compared with unexposed controls. Comparable influence of AgNPs and dissolved Ag on the lipid peroxidation suggests that the dissolved Ag present in the AgNPs suspensions drives the observed effect. Lipid peroxidation is considered a marker of oxidative stress damage^39^. Severe lipid peroxidation could lead to structural change in membranes, impaired membrane fluidity and canals, altered signaling proteins linked to membranes, and increased membrane permeability to ions^40^.

The maximal quantum yield (Fv/Fm), used as a measure for the efficiency of PSII was significantly decreased after 24 h exposure to AgNPs or dissolved Ag, while at 2 h the Fv/Fm were comparable with those of the untreated controls (Fig. 3C). The fold change of Fv/Fm decreased from 0.93 ± 0.02 at 2 h to 0.76 ± 0.16 at 24 h exposure to AgNPs. The electron transport rate of PS II is also affected since it is proportional to the quantum yield^41^. The above finding is consistent with the existing literature showing a significant decrease of the photosynthetic yield of alga *Isochrysis galbana* exposed to high concentrations of AgNPs^42^ as well as phototrophic plankton communities^43^. The inhibition of PSII can be explained by the sensitivity of the photosynthetic machinery to Ag^+^. Indeed, Ag^+^ was shown to bind to thiols of functional proteins, displacing Cu^+^ in the proteins and causing disturbance or inactivation of the photosynthetic electron transport of the photosystem in a green alga *Chlamydomonas reinhardtii*^44^. In addition, about two-fold increase of non-photochemical quenching (NPQ) by both Ag-treatments was observed (Fig. 3D). The fold change of NPQ were 2.34 ± 0.06 and 2.86 ± 0.10 at 2 and 24 h-exposure to AgNPs with respect to untreated controls. NPQ is the degree to which photons are lost in the photosynthetic process, therefore the energy absorbed is divided between the fraction used in photochemistry and that lost non-photochemically. Phytoplankton can regulate the light-harvesting domain, increase the amount of energy dissipated as heat via NPQ, thus protecting the cells from oxidative stress^45^. These results showed that the photosynthetic performance of *P. malhamensis* at 24 h was strongly affected by AgNPs and dissolved Ag.

### Metabolic response of *P. malhamensis* to AgNPs and dissolved Ag

**Figure 4:**
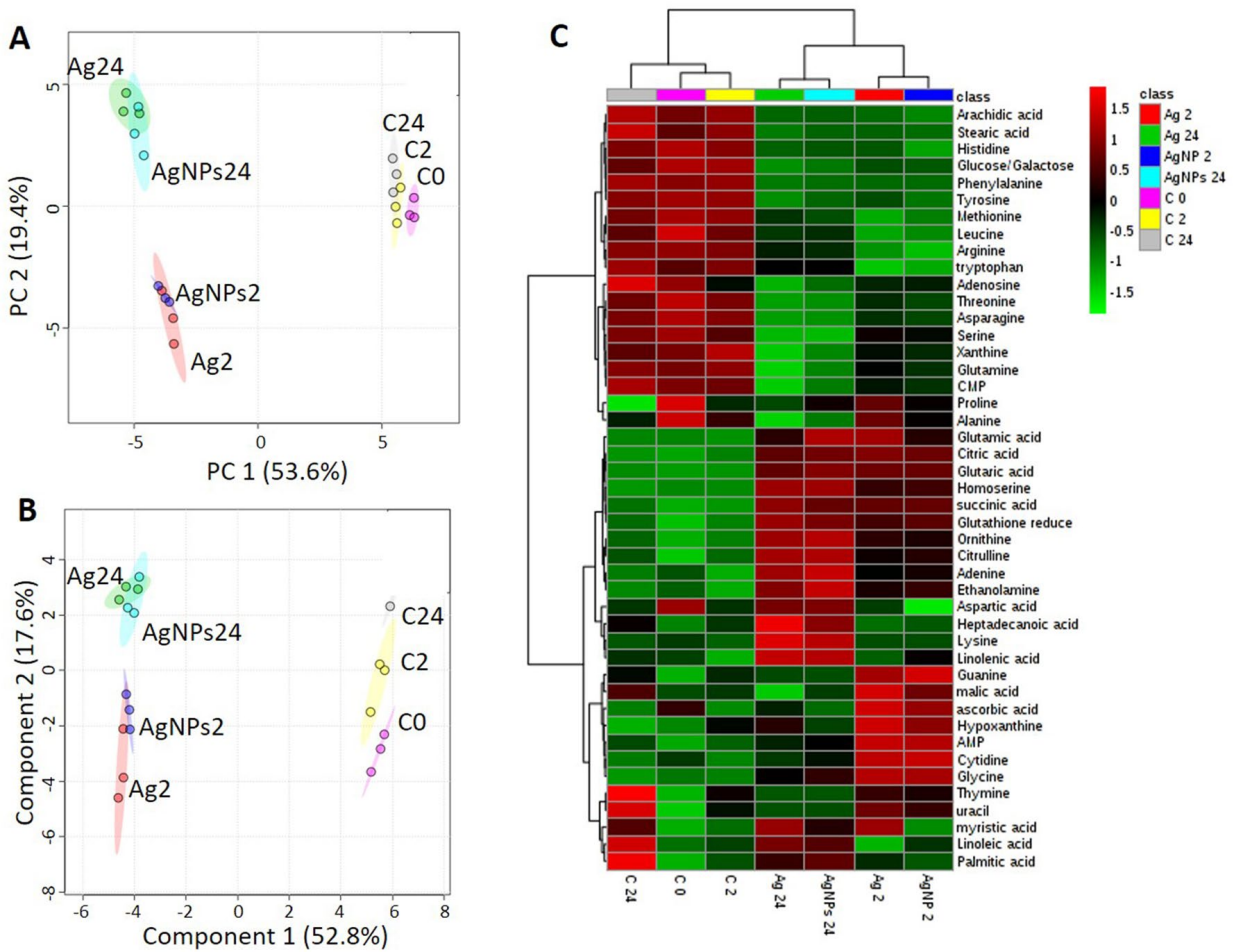
(**A**) Principal component analysis (PCA) and (**B**) partial least-squares discriminate analysis (PLS-DA) score plots of metabolic profiles in *P. malhamensis* treated with AgNPs and dissolved Ag, and untreated controls. (**C**) Clustering metabolites and samples shown in a heat map (Euclidean distance and Ward clustering algorithm). Data were normalized by using probabilistic quotient normalization by untreated control group at time 0 (C0), log transformed and autoscaled. Exposure to 40.7 μg L^−1^AgNO_3_ for 2 h (Ag2) and 24 h (Ag24); exposure to 1 mgL^−1^AgNPs for 2 h (AgNPs2) and for 24 (AgNPs24); C2: unexposed controls at time 2 h; C24: unexposed controls at time 24 h. The score plots and heatmap are generated by MetaboAnalyst 4.0 (https://www.metaboanalyst.ca/)^72^.

A total of 94 metabolites were considered, representing major groups of primary metabolites, including antioxidants, amines, amino acids, organic acids/phenolics, nucleobases/sides/tides, sugars/sugar alcohols and fatty acids. Among them 52 were quantified in different Ag-treatments and controls. A general overview of the treatment clustering was obtained by the unsupervised PCA, supervised PLS-DA and clustering analysis (Fig. 4).

The PCA score plot (Fig. 4A) demonstrated very good separation of both Ag-treatments and unexposed controls, along the first principal component (PC1), which explained 53.6% of the total variance. Excellent separation was found between 2 and 24 h exposures, underlying the importance of tracking the evolution of the metabolic response with time. The PLS-DA score plot (Fig. 4B) confirmed a distinction between Ag-treatments at 2 h and 24 h and untreated samples. However, no noticeable separation was observed between AgNPs and dissolved Ag-treatments for a given exposure time. The responsive metabolites were subsequently discriminated on the basis of VIP score > 1 (Fig. S1).

Twenty-seven important features identified by PLS-DA allowed a good separation between both AgNPs and Ag-treated samples and untreated controls at 2 and 24 h (Fig. S1). Eighteen additional significantly dysregulated metabolites in the Ag-treatments were identified by multivariate analysis (ANOVA, *p* < 0.05, Table S1). The abundances of these 45 responsive metabolites were significantly different between unexposed controls and Ag-treatments for both exposure times. The heat map clustering confirmed a sample grouping of the untreated controls and Ag treatments at 2 and 24 h (Fig. 4C). Five clusters were obtained, corresponding to metabolic perturbation due to Ag-treatments and time progression: Cluster 1 represents the 17 metabolites depleted under the Ag-treatments at 2 and 24 h; Cluster 2 corresponds to 10 metabolites accumulated during the Ag-treatments, to a larger extent at 24 h than at 2 h-exposure; Cluster 3 included 4 metabolites accumulated only at 24 h; Cluster 4 comprises 7 metabolites accumulated only at 2 h-exposure. A more complex pattern was observed for metabolites in Cluster 5.

### Time course of metabolic changes induced by AgNPs and dissolved Ag

#### Amino acid metabolism

**Figure 5:**
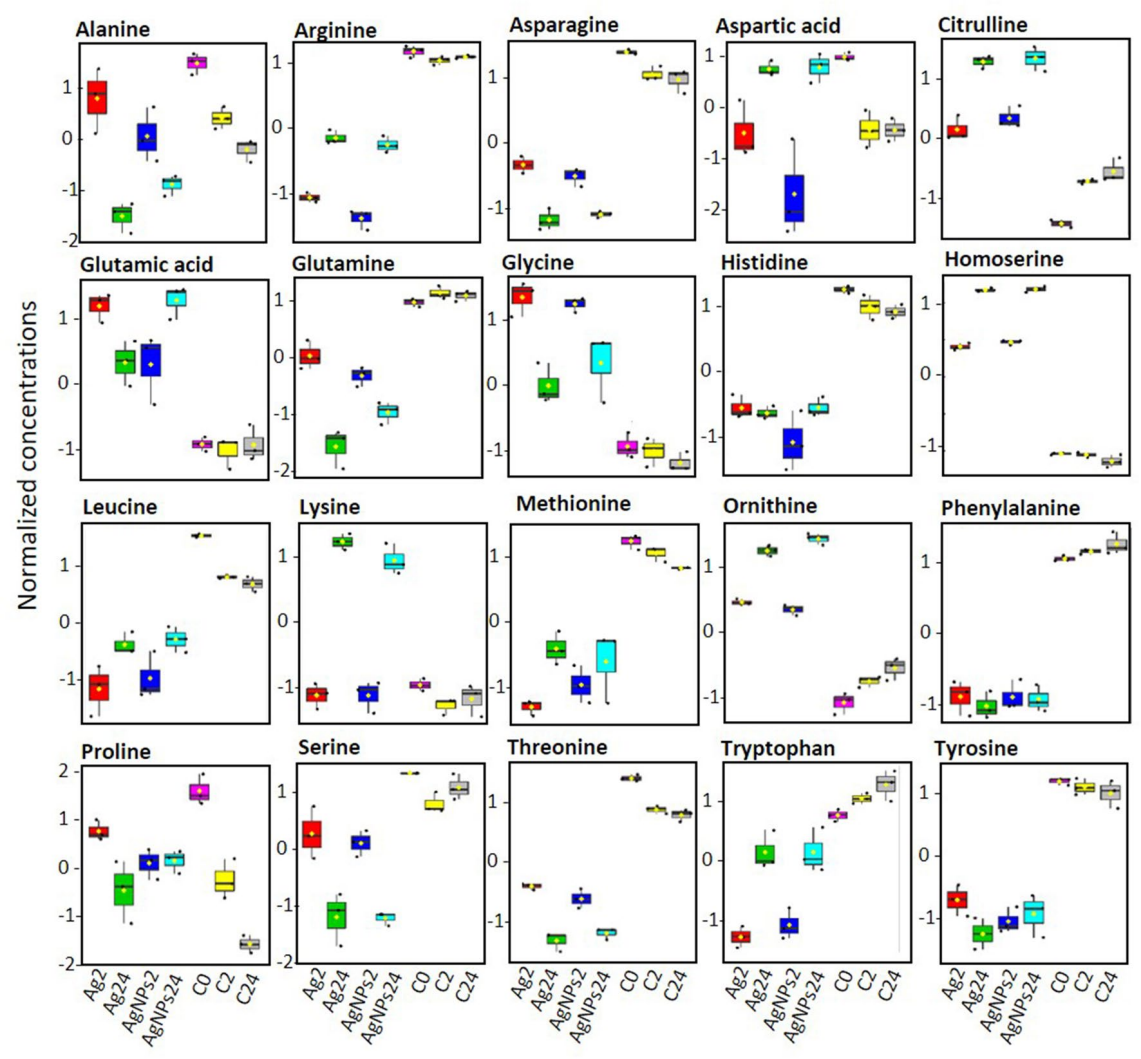
Box plots of relative abundance of amino acids in *P. malhamensis* in Ag-treatments and untreated controls. Data were normalized by using probabilistic quotient normalization by untreated control group at time 0 (C0), log transformed and autoscaled. Exposure to 40.7 μg L^−1^AgNO_3_ for 2 h (Ag2) and 24 h (Ag24); exposure to 1 mg L^−1^AgNPs for 2 h (AgNPs2) and for 24 (AgNPs24); C2: unexposed controls at time 2 h; C24: unexposed controls at time 24 h.

**Figure 6:**
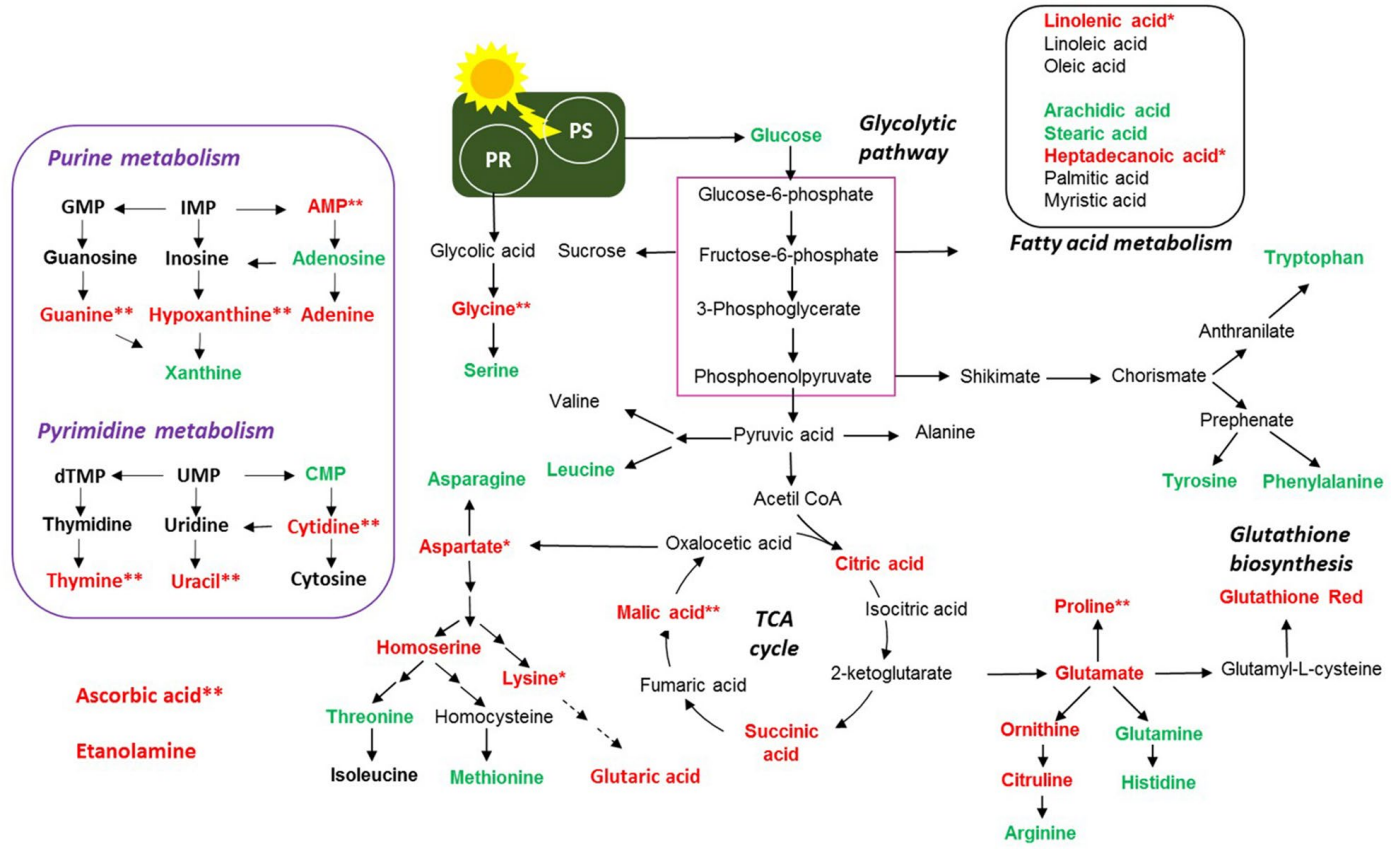
Overview of the proposed perturbations in metabolic pathways in golden-brown alga *P. malhamensis* exposed to 1 mg L^−1^AgNPs and 40.7 μg L^−1^ AgNO_3_, corresponding to the dissolved Ag in AgNPs suspension, for 2 and 24 h. Accumulated and depleted metabolites are present in red and green colors. *denotes perturbations observed at 24 h-exposure only; **denotes perturbations observed at 2 h-exposure only, *PS* photosynthesis, *PR* photorespiration. Only significantly altered metabolites (Supplementary Table S1; Supplementary Fig. S1) were considered. Data were normalized by using probabilistic quotient normalization by untreated control group at time 0 (C0), log transformed and autoscaled.

Exposure of *P. malhamensis* to AgNPs or dissolved Ag resulted in a significant alteration of amino acid metabolism (Figs. 5, 6). Amino acids are important primary metabolites, which are the structural units of the proteins and polypeptides and serve as precursors for the synthesis of other metabolites with multiple functions in algal growth and other biological processes^46,47^.

A significant decrease (*p* < 0.05) in the relative abundance of a number of amino acids (arginine, asparagine, glutamine, histidine, leucine, phenylalanine, serine, threonine, tryptophan and tyrosine) was found in both 2 h and 24 h treatments (Fig. 5), suggesting either a reduction of amino acid synthesis or intensified use of these amino acids in response to Ag-treatment induced stress.

Glutamine and asparagine, two N-rich amino acids involved in nitrate^48^ and ammonium^49^ assimilation in plants were depleted, suggesting that the capacity to acquire nitrogen compounds is lowered. Leucine, a branched chain amino acid serving as an oxidative phosphorylation energy source or as a detoxification pathway^50^ was also significantly decreased (*p* < 0.05) as compared with unexposed controls. Arginine and histidine, positively charged amino acids, were also significantly depleted. Histidine is an amino acid needed for growth and development of algal cells, therefore such depletion revealed the influence of both Ag-treatments on algal growth. In addition, histidine and arginine participate in deamination^47,51^, showing that this process can be also altered. By contrast, the abundance of aspartate, citrulline, glutamic acid, glycine, homoserine, lysine and ornithine increased with both Ag-treatments at 2 and 24 h, suggesting an active defense of *P. malhamensis* against the stress induced by Ag-treatments. For example, glutamic acid is known to serve as a signaling molecule and to play a role in the antioxidant defense in plants^52^. Moreover, the increase of glutamic acid could be related to the detoxication mechanism via induction of synthesis of phytochelatins (PCs), which form stable complexes with metals^53^.

Glycine and serine are part of the photorespiratory glycolate cycle in algae and their ratio is used as an indicator of the photorespiratory activity^54^. The Gly/Ser ratio is significantly increased in the treatments from 1.88 $\pm$ 0.14 for the controls to 4.81 $\pm$ 0.38 and 5.32 $\pm$ 0.52 for 24 h exposures to dissolved Ag and AgNPs, respectively. Interesting, the Gly/Ser ratio also increased with exposure time from 2 to 24 h, for example from 4.61 $\pm$ 0.35 to 5.32 $\pm$ 0.52 for AgNPs (*p* < 0.05). The change from 4.52 $\pm$ 0.32 to 4.81 $\pm$ 0.38 for AgNO_3_ was not statistically significant. For the unexposed controls, this ratio was unchanged over time. These results clearly demonstrated that Ag-treatments induced an increase in photorespiration, probably to produce the energy necessary for the synthesis of different defense components needed to cope with the oxidative stress in a time dependent manner. Photorespiration may provide additional protection against oxidative damage supplying glycine (also enhanced) used for synthesis of the antioxidant glutathione^53^. In fact, photorespiration can serve as an “energy sink, preventing the over-reduction of photosynthetic electron transport chain and photoinhibition”^55^, especially under stressed conditions that lead to reduced rates of photosynthetic CO_2_ assimilation as observed for Ag-treatments. These findings are in line with the reduced photochemical yield and electron transport rate of PS II (Fig. 3C) that was observed at 24 h exposure. Furthermore, serine can be synthesized by a non-photorespiratory pathway^56^, which was also probably perturbed. The accumulated amino acids can also contribute to chelation of Ag^+^ inside the cells^52^. Methionine was significantly decreased by both Ag-treatments. Methionine is a sulfur-containing amino acid, central in many processes of the cellular metabolism, including protein synthesis^57,58^. Methionine is the first amino acid to be translated in protein synthesis by initiating mRNA translation and is the precursor of essential bio-molecules through S-adenosylmethionine^59^. It can be used to provide an estimate of the rate of protein synthesis^60^. Therefore, the depletion of this metabolite suggests a significant reduction of the rate of protein synthesis already at 2 h exposure. This is very important, because it demonstrates how metabolomics can serve to track effects well before they are observed physiologically. Given the high affinity of Ag^+^ to SH-groups, methionine depletion could be also due to its consumption to chelate Ag^+^.

Proline was significantly accumulated after only 2 h exposure to both Ag-treatments. Proline is known to play an important role in osmo- and redox-regulation, Cd and Zn chelation, and scavenging of free radicals induced as a result of Cd, Cu, Hg and Zn exposure in plants^52,60^. Hence, it can be hypothesized that the accumulation of proline upon exposure to AgNPs and dissolved Ag could serve as one of the first defense lines to cope with Ag-induced oxidative stress at 2 h exposure. Proline accumulation could be a result of an accelerated synthesis, release from macromolecules or inhibition of its degradation, therefore one or more of these processes could be affected by the Ag-treatments ^52^.

Overall, significant changes in the amino acid levels with Ag treatments and over time were found, suggesting a perturbation of N-metabolism. In addition, an SH- containing amino acid, methionine, is a component of S-metabolism in algae^62^ and its depletion suggested that S-assimilation is disturbed.

#### Nucleobase/tide/side metabolism

Exposure to AgNPs and dissolved Ag altered the nucleic acids metabolism of *P. malhamensis* (Fig. 6, Supplementary Fig. S2). The metabolism of both purine and pyrimidine derivatives was affected. For example, a significant depletion of cytidine monophosphate (CMP), a cytosine-based nucleotide was accompanied by an accumulation of cytidine, suggesting a perturbation of the pyrimidine metabolism^63,64^. Similarly, guanine, hypoxanthine and adenine were accumulated in both Ag-treatments, indicating that purine metabolism was also altered^63,64^. Such nucleobase accumulation could be related to a nucleoside degradation due to the Ag-treatments. Nevertheless, no significant changes in the abundance of other nucleobases/tides/sides such as guanosine, inosine, thymidine, thymine, uridine and uracil were observed.

#### Sugars metabolism

Glucose is a primary product of photosynthesis, and depletion of glucose revealed that photosynthesis could be decreased (Fig. 6) and the alga capability to fix C was reduced in response to AgNPs and dissolved Ag. Photosynthesis is a fundamental process, converting CO_2_ to organic carbon available to phytoplankton cells. The decrease of the abundance of glucose upon Ag-treatments was more pronounced in AgNPs treatment and at 24 h exposure. The above finding is consistent with the observed inhibition of the photochemical yield at 24 h exposure (Fig. 3C) and the two-fold increase of NPQ by AgNPs and dissolved Ag (Fig. 3D). This observation agrees with the accelerated photorespiration observed during both Ag-treatments. In addition, a decrease in glucose concentration and the increase of TCA metabolites could also be related with induction of the mitochondrial activity^65^.

#### Fatty acids metabolism

The unsaturated fatty acid linolenic acid (C 9,12,15 double bonds) accumulated more after 24 h exposure to AgNPs and dissolved Ag, while linoleic (C 9,12 double bonds) and oleic (C 9 double bond) acids were unchanged. In parallel, a significant depletion of the saturated acids—arachidic (C20 strait chain), stearic (C18 strait chain) acids and to a lower degree heptadecanoic acid (C17 strait chain)—was observed (Fig. 6, Supplementary Fig. S3). Other saturated fatty acids, such as palmitic acid (C16 strait chain), were unaffected. These metabolite changes indicate that Ag-treatment induced unsaturation of the lipid membranes and altered the composition and integrity of lipid membranes. Indeed, this finding agrees with the strong lipid peroxidation observed in both Ag-treatments (Fig. 3B). A perturbation of the metabolism of fatty acids can also change the energy budget^66^. An alteration of the fatty acid composition is a common observation under stress, and algae have been shown to adjust the degree of unsaturation of the membrane to combat oxidative stress induced by toxic metals^67^. Furthermore, plants could adjust the composition of fatty acids in the membrane to rebuild membrane integrity, as was shown for cucumber leaves exposed to AgNPs and dissolved Ag^5^. Treatments with AgNPs and dissolved Ag were also shown to reduce monounsaturated and polyunsaturated fatty acids of green microalgae *Chlorella vulgaris*^68^.

#### Carboxylic acid metabolism and antioxidants

Citric acid and succinic acid, intermediates in the tricarboxylic acid (TCA) cycle significantly accumulated in both Ag-treatments after 2 h and 24 h (Fig. 6, Supplementary Fig. S4). With the exception of the 2 h AgNPs treatment, malic acid levels in *P. malamensis* increased significantly (*p* < 0.05) in all other treatments. This finding clearly showed that the TCA cycle, which is key metabolic pathway that connects carbohydrate, fatty acids, and protein metabolism, was accelerated. The TCA cycle is the core of the cell’s respiratory machinery; it is likely that algae accelerate respiration to produce energy necessary for the manufacture of defense compounds needed to address oxidative stress. This observation is consistent with the increased ratios of the Gly/Ser (see “Amino acid metabolism”). Exposure to AgNPs and dissolved Ag led to the significant increase in the concentrations of two antioxidant molecules: ascorbic acid and reduced glutathione (GSH) (Fig. 6). The accumulation of the ascorbic acid was more pronounced at 2 h exposure than in 24 h, while the increase in the GSH was greater in 24 h treatments. The results clearly indicate that the antioxidant defense system of *P. malamensis* was activated by Ag-treatments, which resulted in an accumulation of the ROS-scavenging metabolites to cope with the enhanced generation of ROS (Fig. 3A). In addition, GSH is a precursor of PCs which is activated by different toxic metals including Ag^69^.

Taken together, the decrease of fatty acids, the increase of the antioxidants level, such as GSH, and GSH–related amino acids glycine and glutamate, suggest that the AgNPs and dissolved Ag triggered an excessive generation of ROS, in agreement with experimentally observed oxidative stress (Fig. 3A) and lipid peroxidation (Fig. 3B). These observations corroborate the significant accumulation of glutaric acid after Ag-treatments, since glutaric acid, is a product of the fatty acid and lysine degradation metabolism.

### Metabolic response specific to AgNPs

To discriminate between metabolic perturbations induced by AgNPs and dissolved Ag, separate analyses of the respective data sets for 2 h and 24 h were conducted (Supplementary Fig. S5, Supplementary Table S2; Supplementary Fig. S6, Supplementary Table S3). For both 2 h an 24 h exposures, the PCA score plot (Fig. S5A, S6A) confirmed good separation between AgNPs, dissolved Ag and unexposed controls. A supervised PLS-DA score plot (Supplementary Figs. S5B, S6B) resulted in clear separation between exposures to AgNPs and dissolved Ag. The responsive metabolites, isolated on the basis of VIP score > 1 included 33 molecules for 2 h exposure (Supplementary Fig. S5C) and 34 molecules for 24 h exposure (Supplementary Fig. S6C). Metabolites commonly or specifically perturbed at 2 h and 24 h-exposure which discriminate AgNPs and dissolved Ag responses are present in Supplementary Fig. S7.

No specific metabolites were affected in response to AgNPs only. However, some metabolites were either accumulated or depleted to a larger extent after exposure to AgNPs as compared with dissolved Ag. As the experiment was designed to distinguish the responses in the presence of AgNPs and AgNO_3_ corresponding to the dissolved Ag in the particle suspensions, these metabolic changes showed an important contribution of the particulate form to the overall response. At 2 h exposure to AgNPs adenine, glycine and homoserine were upregulated more strongly (*p* < 0.05) than in dissolved Ag-exposure; while arginine, asparagine, glutamine, histidine, threonine, tyrosine and xanthine were depleted to a larger extent (*p* < 0.05), indicating the important contribution of AgNPs to the perturbation of amino acid metabolism. CMP was depleted, while cytidine abundance increased more strongly in AgNPs than in the dissolved Ag-treatments, showing significant involvement of AgNPs in the perturbation of pyrimidine metabolism. A higher accumulation of GSH was also found in AgNPs treatments at 2 h, pointing out the important role of AgNPs in induction of oxidative stress, which corroborate the results of the cellular ROS generation (Fig. 3A). A bigger accumulation of succinic acid in AgNPs than in dissolved Ag-treatment indicated a significant role of the AgNPs in TCA cycle perturbation. Glucose levels decreased more after 2 h exposure to AgNPs, in agreement with the photosynthesis inhibition results. Arachidic acid depletion was also larger in AgNPs than dissolved Ag-treatments (Supplementary Fig. S5). At 24 h exposure to AgNPs arginine and methionine decrease was stronger; whereas aspartic acid, citrulline, glycine, glutamic acid, glutaric acid, homoserine, ornithine, proline increase was more pronounced. Adenine and AMP were accumulated to a larger extent, suggesting stronger impact of the AgNPs on the purine metabolism (Supplementary Fig. S6). At 24 h exposure, no significant difference was found in the AgNPs and dissolved Ag-induced perturbation in the TCA cycle, fatty acid metabolism and GSH concentrations.

## Conclusion

The present study for the first time revealed the metabolic perturbations induced by AgNPs following their uptake and accumulation in the food vacuoles of golden-brown alga *P. malhamensis.* Results of targeted metabolomics demonstrated that the exposure to AgNPs and dissolved Ag resulted in time-dependent perturbation of the concentration of metabolites involved in various metabolic pathways involving amino acids, nucleotides, fatty acids, TCA, antioxidants, photosynthesis and photorespiration. Even though the intensity of the responses differed with exposure time and type of Ag in the treatments, the observed metabolic perturbations were common for the particulate and dissolved forms. The results suggest that dissolved Ag released by AgNPs are the major toxicity driver, even though AgNPs are internalized in the food vacuoles. However, AgNPs play an important role in the perturbation of amino acid metabolism, TCA cycle and oxidative stress, in particularly at 2 h exposure. Their role diminished at 24 h since the AgNPs in the food vacuoles aggregated substantially by then, and thus released less Ag^+^. The metabolomic perturbations were reflected in the physiological responses such as increased cellular ROS generation (more pronounced in AgNPs, treatments); lipid peroxidation in *P. malhamensis*, and decreases of the photosynthetic efficiency for both Ag-treatments with time. This study demonstrates the value of metabolomics as a tool for understanding the molecular basis for these metabolic and physiological changes, and to detect early on metabolic changes that can later express themselves physiologically.

## Methods

### Characterization of AgNPs in exposure medium

Citrate-coated spherical AgNPs (BioPure) with a primary size of 20 nm at 1.01 g Ag L^−1^ (2 × 10^13^ NP mL^−1^) in 2 mM citrate, were purchased from Nanocomposix (San Diego, USA). Silver nitrate (AgNO_3_) were purchased from Sigma-Aldrich. The characteristics of the AgNPs in the stock suspension as provided by the manufacturer can be found in Supplementary Fig. S8. AgNP suspensions in the exposure medium was characterized in terms of dissolution and aggregation at 2 h and 24 h. The hydrodynamic diameters and their distributions, and the zeta potential (ZP) were measured using a Malvern Zetasizer Nano (Malvern Instruments Inc, UK). The size and morphology of the AgNPs in the exposure medium were determined by transmission electronic microscopy (TEM) (FEI Tecnai™ G2 Sphera, FEI Company, USA). The percentage of dissolved silver in the suspensions of AgNPs at 2 and 24 h was assessed by ultracentrifugation and analysis of the dissolved Ag concentration in the supernatant by inductively coupled plasma mass spectrometry (ICP-MS, Agilent 7700x, Basel, Switzerland) as detailed in the SI.

### Bioassays with *P. malhamensis*

*Poterioochromonas malhamensis* (CCAC 3498 strain, Cologne Biocenter, Germany) was grown in a modified Waris-H medium (Supplementary Table S4) at 25 °C with a light illumination of 5 µmol photons m^−2^ s^−1^ in a 12:12 light–dark cycle in the specialized incubator (MIR 253, Sanyo, Japan). The cells were harvested and re-suspended in the modified Waris-H medium enriched with AgNPs to a cell density of 10^6^ cells mL^−1^. The suspension of 1 mgL^−1^ AgNPs in the exposure medium were prepared by 1000 times dilution of the stock suspension (1g L^−1^) and homogenization by vortex for 45 s before use. Uptake, physiological and metabolic perturbations were determined at 2 and 24 h. To distinguish between the effects of AgNPs and dissolved Ag present in the AgNPs suspensions on the algal metabolism and physiological effects, the experiments were performed in the presence of 1 mg L^−1^ AgNPs and 40.7 μg L^−1^ AgNO_3_ at 2 and 24 h-exposure. The concentration of AgNO_3_ corresponds to the dissolved Ag in the suspensions of 1 mgL^−1^AgNPs. The choice of AgNPs concentrations was made based on some preliminary bioassays (Supplementary Figs. S9–S12).

### Ag cellular burden and AgNPs internalization

To measure the total Ag burden upon AgNPs and AgNO_3_ exposure, the exposed cell suspensions were centrifuged at 1000*g* for 10 min. The pellets containing algae were rinsed twice with Ag free Waris-H medium to remove all loosely bound particles from algal surface and then digested in ultrapure HNO_3_ overnight at 90 °C in an oven, diluted with MilliQ water and analyzed by ICP-MS (Agilent 7700x, Basel, Switzerland).

To determine the cellular distribution of AgNPs in *P. melhamensis*, the cells were fixed with 2% glutaraldehyde and 4% formaldehyde overnight at 4 °C. After post-fixation staining with 1% osmium tetroxide, the cells were embedded in epoxy resin. Ultrathin section (80 nm) were obtained with an ultramicrotome (Leica, Bannockburn, IL, USA). These sections were stained with 2% uranyl acetate solution and plumbic citrate, and then placed on 200 mesh copper grids. Morphological characteristics of cells and the distribution of particles within the cells were analyzed with Transmission Electron Microscopy (TEM, FEI Tecnai™ G2 Sphera, FEI Company, USA) operated at 80 kV. Energy dispersive X-ray detector (AMETEK Inc. Germany) was used to carry out the chemical analysis. The measurement and analyses were operated by the software Genesis version 6.255.

### Physiological effects of AgNPs and AgNO_3_

The influence of AgNPs and AgNO_3_ on cell growth, generation of ROS, lipid peroxidation and photosynthetic activity was assessed as detailed in the SI. Briefly, cellular ROS generation was followed by BD Accuri C6 flow cytometer (BD Biosciences, San Jose, CA) using the CellROX@Green stain (Life Technologies Europe B.V., Zug, Switzerland) following the procedure adapted from^70^. Unexposed algae were used as negative control, while algae exposed to 2.5 mM H_2_O_2_ for 20 min were used as positive control. Lipid peroxidation was assessed by malondialdehyde (MAD) kit (Sigma-Aldrich. St Louis, USA) and spectrophotometry detection. Changes in the photosynthetic activity of *P. malhamensis* during Ag-treatments were followed using a Multiple Excitation Wavelength Chlorophyll Fluorescence Analyzer (Multi-Color-PAM, Walz, Germany). Maximal fluorescence yield of the photosystem II, Fm, and maximal variable fluorescence, Fv (Fv/Fm) and non-photochemical quenching (NPQ), corresponding to the dissipation of excess energy as heat loss from PSII, were measured after 2 h and 24 h exposure following 20 min of dark acclimation. These parameters are well-known indicators for alteration of photosynthetic activity by different biotic and abiotic stressors^71^. Results are present in Supplementary Fig. S12.

Statistically significant differences of the percentage of affected cells by AgNPs and AgNO_3_ were analyzed using a two-way analysis of variance (ANOVA) followed by a Sidak’s multiple comparisons test (Graphpad Prism 6, Graphpad Sofeware Inc., San Diego, CA, USA).

### LC–MS targeted metabolomics

Metabolic changes in *P. malhamensis* exposed to 1 mg L^−1^ AgNPs or 40.7 μg L^−1^AgNO_3_ for 2 and 24 h were determined by LC–MS targeted metabolomics. Untreated algae were used as control. Control and exposed *P. malhamensis* were sampled at 0 h (t0, beginning of the exposure), 2 h and 24 h to assess the variation in metabolite levels during the cell development cycle. At the end of the exposure, the cells were put in liquid nitrogen to stop metabolic activity, then frozen at − 80 °C for 24 h and freeze-dried. Different metabolites, including antioxidants, amines, amino acids, organic acids/phenolics, nucleobase/side/tide, sugar/sugar alcohols and fatty acids were extracted in 80% methanol containing 2% formic acid following previously developed methodology^8,9^. Targeted analyses of the metabolites were performed using Agilent 6470 liquid chromatography triple quadrupole mass spectrometer according to previously established methods with MS parameters, as shown in Supplementary Table S6^4,8,9^.

Statistical analysis of the metabolomics data was performed by using MetaboAnalyst 4.0^72^. Data were normalized using the probabilistic quotient normalization by the unexposed control group at t0, then log-normalized and autoscaled. Exploratory data analysis was performed with one-way analysis of variance (ANOVA) followed by a Fisher's least significant difference method (Fisher's LSD) with *p* value threshold of 0.05. Unsupervised Principal Component Analysis (PCA) and supervised Partial Least Squares—Discriminant Analysis (PLS-DA) were employed to cluster different treatments. Variables with a importance in the projection (VIP) greater than 1 were regarded as significant and responsible for group separation discriminating metabolites^73^. The changes in the abundance of measured metabolites are illustrated in the boxplots, generated by MetaboAnalyst 4.0. The plots were treated to increase the level size and axis titles and thus improve the readability.

## Supplementary information

Supplementary Information.

## Data Availability

All data supporting the findings of this study are available within the article and its supplementary information file.
