# Supplementary material for: Metabolomics for early detection of stress in freshwater alga Poterioochromonas malhamensis exposed to silver nanoparticles
Source: Sci Rep. 2020 Nov 25;10:20563. doi: 10.1038/s41598-020-77521-0 (PMC7689461; doi:10.1038/s41598-020-77521-0)
Supplement: Supplementary file 1 — Supplementary Information. [file 41598_2020_77521_MOESM1_ESM.pdf]

Supplementary information

Metabolomics for early detection of stress in freshwater alga *Poterioochromonas malhamensis* exposed to silver nanoparticles

Wei Liu[a], Sanghamitra Majumdar[b], Weiwei Li[b], Arturo A. Keller[b], Vera I. Slaveykova[a,*]

[a]University of Geneva, Faculty of Sciences, Earth and Environment Sciences, Department F.-A. Forel for Environmental and Aquatic Sciences, Environmental Biogeochemistry and Ecotoxicology, Uni Carl Vogt, 66 Blvd Carl-Vogt, CH 1211 Geneva, Switzerland

[b]Bren School of Environmental Science & Management, University of California, Santa Barbara, California 93106-5131, United States

*corresponding author: vera.slaveykova@unige.ch

Content

1. Metabolic perturbations induced by AgNPs and dissolved Ag
2. Different groups of metabolites identified by cluster analysis
3. AgNPs and dissolved Ag induced changes in abundance of nucleotide/side metabolites
4. AgNPs and dissolved Ag induced changes in abundance of fatty acids metabolites
5. AgNPs and dissolved Ag induced changes in abundance of carbocyclic acids metabolites
6. Metabolic perturbations induced by 2h-exposure to AgNPs and dissolved Ag
7. Metabolic perturbations induced by 24h-exposure to AgNPs and dissolved Ag
8. Metabolites common and specific for the two exposure times
9. Characteristics of stock suspension of AgNPs
10. Composition of the Waris-H medium
11. Determination of AgNPs dissolution in the exposure medium
12. Silver cellular burden of *P. malhamensis* exposed to AgNPs for 2 and 24h
13. Effect of AgNPs and dissolved Ag on algal growth
14. Determination of the cellular ROS upon AgNPs and dissolved Ag exposure
15. Determination of the lipid peroxidation upon AgNPs and dissolved Ag exposure
16. Determination of photosynthetic activity upon AgNPs and dissolved Ag exposure
17. Metabolites and MS parameters for LC-MS targeted metabolomics

1. Metabolic perturbations induced by AgNPs and dissolved Ag

Table S1. Important features identified by One-way ANOVA and Fisher's post-hoc analysis in *P. malhamensis* exposed to 40.7 μgL^{-1} $AgNO_3$; and 1 mgL^{-1} AgNPs and unexposed controls at 2 and 24h. Data were normalized with respect to unexposed control at time 0 (C0).

Metabolite	f.value	p.value	-LOG10(p)	FDR	Fisher's LSD
Homoserine	1688.3	3.4258e-19	18.465	1.7129e-17	Ag 24 - Ag 2; AgNPs 24 - Ag 2; Ag 2 - C 0; Ag 2 - C 2; Ag 2 - C 24; Ag 24 - AgNP 2; Ag 24 - C 0; Ag 24 - C 2; Ag 24 - C 24; AgNPs 24 - AgNP 2; AgNP 2 - C 0; AgNP 2 - C 2; AgNP 2 - C 24; AgNPs 24 - C 0; AgNPs 24 - C 2; AgNPs 24 - C 24; C 0 - C 24; C 2 - C 24
Arginine	335.41	2.6716e-14	13.573	4.9115e-13	Ag 24 - Ag 2; Ag 2 - AgNP 2; AgNPs 24 - Ag 2; C 0 - Ag 2; C 2 - Ag 2; C 24 - Ag 2; Ag 24 - AgNP 2; C 0 - Ag 24; C 2 - Ag 24; C 24 - Ag 24; AgNPs 24 - AgNP 2; C 0 - AgNP 2; C 2 - AgNP 2; C 24 - AgNP 2; C 0 - AgNPs 24; C 2 - AgNPs 24; C 24 - AgNPs 24
Citric acid	330.7	2.9469e-14	13.531	4.9115e-13	Ag 2 - Ag 24; Ag 2 - C 0; Ag 2 - C 2; Ag 2 - C 24; Ag 24 - C 0; Ag 24 - C 2; Ag 24 - C 24; AgNP 2 - C 0; AgNP 2 - C 2; AgNP 2 - C 24; AgNPs 24 - C 0; AgNPs 24 - C 2; AgNPs 24 - C 24; C 2 - C 0
Threonine	303.63	5.3286e-14	13.273	6.6608e-13	Ag 2 - Ag 24; Ag 2 - AgNP 2; Ag 2 - AgNPs 24; C 0 - Ag 2; C 2 - Ag 2; C 24 - Ag 2; AgNP 2 - Ag 24; C 0 - Ag 24; C 2 - Ag 24; C 24 - Ag 24; AgNP 2 - AgNPs 24; C 0 - AgNP 2; C 2 - AgNP 2; C 24 - AgNP 2; C 0 - AgNPs 24; C 2 - AgNPs 24; C 24 - AgNPs 24; C 0 - C 2; C 0 - C 24
Ornithine	236.86	2.9751e-13	12.526	2.9751e-12	Ag 24 - Ag 2; AgNPs 24 - Ag 2; Ag 2 - C 0; Ag 2 - C 2; Ag 2 - C 24; Ag 24 - AgNP 2; Ag 24 - C 0; Ag 24 - C 2; Ag 24 - C 24; AgNPs 24 - AgNP 2; AgNP 2 - C 0; AgNP 2 - C 2; AgNP 2 - C 24; AgNPs 24 - C 0; AgNPs 24 - C 2; AgNPs 24 - C 24; C 2 - C 0; C 24 - C 0; C 24 - C 2
Glutaric acid	222.3	4.6117e-13	12.336	3.843e-12	Ag 2 - C 0; Ag 2 - C 2; Ag 2 - C 24; AgNPs 24 - Ag 24; Ag 24 - C 0; Ag 24 - C 2; Ag 24 - C 24; AgNP 2 - C 0; AgNP 2 - C 2; AgNP 2 - C 24; AgNPs 24 - C 0; AgNPs 24 - C 2; AgNPs 24 - C 24
Asparagine	209.17	7.0235e-13	12.153	5.0168e-12	Ag 2 - Ag 24; Ag 2 - AgNPs 24; C 0 - Ag 2; C 2 - Ag 2; C 24 - Ag 2; AgNP 2 - Ag 24; C 0 - Ag 24; C 2 - Ag 24; C 24 - Ag 24; AgNP 2 - AgNPs 24; C 0 - AgNP 2; C 2 - AgNP 2; C 24 - AgNP 2; C 0 - AgNPs 24; C 2 - AgNPs 24; C 24 - AgNPs 24; C 0 - C 2; C 0 - C 24
AMP	140.9	1.0651e-11	10.973	6.657e-11	Ag 2 - Ag 24; Ag 2 - AgNPs 24; Ag 2 - C 0; Ag 2 - C 2; Ag 2 - C 24; AgNP 2 - Ag 24; Ag 24 - C 0; Ag 24 - C 2; Ag 24 - C 24; AgNP 2 - AgNPs 24; AgNP 2 - C 0; AgNP 2 - C 2; AgNP 2 - C 24; AgNPs 24 - C 0; AgNPs 24 - C 2; AgNPs 24 - C 24; C 2 - C 0; C 24 - C 0
Phenylalanine	128.58	1.993e-11	10.7	1.0834e-10	C 0 - Ag 2; C 2 - Ag 2; C 24 - Ag 2; C 0 - Ag 24; C 2 - Ag 24; C 24 - Ag 24; C 0 - AgNP 2; C 2 - AgNP 2; C 24 - AgNP 2; C 0 - AgNPs 24; C 2 - AgNPs 24; C 24 - AgNPs 24
Citrulline	127.02	2.1667e-11	10.664	1.0834e-10	Ag 24 - Ag 2; AgNPs 24 - Ag 2; Ag 2 - C 0; Ag 2 - C 2; Ag 2 - C 24; Ag 24 - AgNP 2; Ag 24 - C 0; Ag 24 - C 2; Ag 24 - C 24; AgNPs 24 - AgNP 2; AgNP 2 - C 0; AgNP 2 - C 2; AgNP 2 - C 24; AgNPs 24 - C 0; AgNPs 24 - C 2; AgNPs 24 - C 24; C 2 - C 0; C 24 - C 0
Adenine	113.51	4.6724e-11	10.33	2.1238e-10	Ag 24 - Ag 2; AgNPs 24 - Ag 2; Ag 2 - C 0; Ag 2 - C 2; Ag 2 - C 24; Ag 24 - AgNP 2; AgNPs 24 - Ag 24; Ag 24 - C 0; Ag 24 - C 2; Ag 24 - C 24; AgNPs 24 - AgNP 2; AgNP 2 - C 0; AgNP 2 - C 2; AgNP 2 - C 24; AgNPs 24 - C 0; AgNPs 24 - C 2; AgNPs 24 - C 24; C 0 - C 2; C 0 - C 24; C 24 - C 2
Arachidic acid	102.66	9.2656e-11	10.033	3.8607e-10	C 0 - Ag 2; C 2 - Ag 2; C 24 - Ag 2; C 0 - Ag 24; C 2 - Ag 24; C 24 - Ag 24; C 0 - AgNP 2; C 2 - AgNP 2; C 24 - AgNP 2; C 0 - AgNPs 24; C 2 - AgNPs 24; C 24 - AgNPs 24; C 24 - C 0

Lysine	99.698	1.1309e-10	9.9466	4.3498e-10	Ag 24 - Ag 2; AgNPs 24 - Ag 2; Ag 24 - AgNP 2; Ag 24 - C 0; Ag 24 - C 2; Ag 24 - C 24; AgNPs 24 - AgNP 2; AgNPs 24 - C 0; AgNPs 24 - C 2; AgNPs 24 - C 24
Xanthine	92.533	1.877e-10	9.7265	6.7036e-10	Ag 2 - Ag 24; Ag 2 - AgNPs 24; C 0 - Ag 2; C 2 - Ag 2; C 24 - Ag 2; AgNP 2 - Ag 24; AgNPs 24 - Ag 24; C 0 - Ag 24; C 2 - Ag 24; C 24 - Ag 24; AgNP 2 - AgNPs 24; C 0 - AgNP 2; C 2 - AgNP 2; C 24 - AgNP 2; C 0 - AgNPs 24; C 2 - AgNPs 24; C 24 - AgNPs 24; C 2 - C 0; C 2 - C 24
Glutamine	87.246	2.7967e-10	9.5534	9.3224e-10	Ag 2 - Ag 24; Ag 2 - AgNP 2; Ag 2 - AgNPs 24; C 0 - Ag 2; C 2 - Ag 2; C 24 - Ag 2; AgNP 2 - Ag 24; AgNPs 24 - Ag 24; C 0 - Ag 24; C 2 - Ag 24; C 24 - Ag 24; AgNP 2 - AgNPs 24; C 0 - AgNP 2; C 2 - AgNP 2; C 24 - AgNP 2; C 0 - AgNPs 24; C 2 - AgNPs 24; C 24 - AgNPs 24
Cytidine	82.499	4.0838e-10	9.3889	1.2762e-09	Ag 2 - Ag 24; Ag 2 - AgNPs 24; Ag 2 - C 0; Ag 2 - C 2; Ag 2 - C 24; AgNP 2 - Ag 24; Ag 24 - C 2; Ag 24 - C 24; AgNP 2 - AgNPs 24; AgNP 2 - C 0; AgNP 2 - C 2; AgNP 2 - C 24; AgNPs 24 - C 2; AgNPs 24 - C 24; C 0 - C 2; C 0 - C 24
Tyrosine	74.57	8.0754e-10	9.0928	2.3751e-09	Ag 2 - Ag 24; C 0 - Ag 2; C 2 - Ag 2; C 24 - Ag 2; C 0 - Ag 24; C 2 - Ag 24; C 24 - Ag 24; C 0 - AgNP 2; C 2 - AgNP 2; C 24 - AgNP 2; C 0 - AgNPs 24; C 2 - AgNPs 24; C 24 - AgNPs 24
Stearic acid	68.881	1.3769e-09	8.8611	3.8247e-09	C 0 - Ag 2; C 2 - Ag 2; C 24 - Ag 2; C 0 - Ag 24; C 2 - Ag 24; C 24 - Ag 24; C 0 - AgNP 2; C 2 - AgNP 2; C 24 - AgNP 2; C 0 - AgNPs 24; C 2 - AgNPs 24; C 24 - AgNPs 24; C 24 - C 0; C 24 - C 2
Succinic acid	61.495	2.9433e-09	8.5312	7.6683e-09	Ag 2 - C 0; Ag 2 - C 2; Ag 2 - C 24; Ag 24 - C 0; Ag 24 - C 2; Ag 24 - C 24; AgNP 2 - C 0; AgNP 2 - C 2; AgNP 2 - C 24; AgNPs 24 - C 0; AgNPs 24 - C 2; AgNPs 24 - C 24; C 24 - C 0
Histidine	61.116	3.0673e-09	8.5132	7.6683e-09	Ag 2 - AgNP 2; C 0 - Ag 2; C 2 - Ag 2; C 24 - Ag 2; Ag 24 - AgNP 2; C 0 - Ag 24; C 2 - Ag 24; C 24 - Ag 24; AgNPs 24 - AgNP 2; C 0 - AgNP 2; C 2 - AgNP 2; C 24 - AgNP 2; C 0 - AgNPs 24; C 2 - AgNPs 24; C 24 - AgNPs 24
Ethanolamine	56.573	5.1332e-09	8.2896	1.2222e-08	Ag 24 - Ag 2; AgNPs 24 - Ag 2; Ag 2 - C 0; Ag 2 - C 2; Ag 2 - C 24; Ag 24 - AgNP 2; AgNPs 24 - Ag 24; Ag 24 - C 0; Ag 24 - C 2; Ag 24 - C 24; AgNPs 24 - AgNP 2; AgNP 2 - C 0; AgNP 2 - C 2; AgNP 2 - C 24; AgNPs 24 - C 0; AgNPs 24 - C 2; AgNPs 24 - C 24; C 0 - C 2
Glutathione<br>reduced	55.418	5.8879e-09	8.23	1.3382e-08	Ag 24 - Ag 2; Ag 2 - C 0; Ag 2 - C 2; Ag 2 - C 24; Ag 24 - AgNP 2; Ag 24 - C 0; Ag 24 - C 2; Ag 24 - C 24; AgNP 2 - C 0; AgNP 2 - C 2; AgNP 2 - C 24; AgNPs 24 - C 0; AgNPs 24 - C 2; AgNPs 24 - C 24; C 2 - C 0; C 24 - C 0
tryptophan	49.076	1.3173e-08	7.8803	2.8637e-08	Ag 24 - Ag 2; AgNPs 24 - Ag 2; C 0 - Ag 2; C 2 - Ag 2; C 24 - Ag 2; Ag 24 - AgNP 2; C 0 - Ag 24; C 2 - Ag 24; C 24 - Ag 24; AgNPs 24 - AgNP 2; C 0 - AgNP 2; C 2 - AgNP 2; C 24 - AgNP 2; C 0 - AgNPs 24; C 2 - AgNPs 24; C 24 - AgNPs 24; C 24 - C 0
Methionine	45.901	2.0468e-08	7.6889	4.2642e-08	Ag 24 - Ag 2; AgNPs 24 - Ag 2; C 0 - Ag 2; C 2 - Ag 2; C 24 - Ag 2; Ag 24 - AgNP 2; C 0 - Ag 24; C 2 - Ag 24; C 24 - Ag 24; C 0 - AgNP 2; C 2 - AgNP 2; C 24 - AgNP 2; C 0 - AgNPs 24; C 2 - AgNPs 24; C 24 - AgNPs 24
Leucine	43.846	2.7654e-08	7.5582	5.5308e-08	Ag 24 - Ag 2; AgNPs 24 - Ag 2; C 0 - Ag 2; C 2 - Ag 2; C 24 - Ag 2; Ag 24 - AgNP 2; C 0 - Ag 24; C 2 - Ag 24; C 24 - Ag 24; AgNPs 24 - AgNP 2; C 0 - AgNP 2; C 2 - AgNP 2; C 24 - AgNP 2; C 0 - AgNPs 24; C 2 - AgNPs 24; C 24 - AgNPs 24; C 0 - C 2; C 0 - C 24
Glycine	42.299	3.4989e-08	7.4561	6.5325e-08	Ag 2 - Ag 24; Ag 2 - AgNPs 24; Ag 2 - C 0; Ag 2 - C 2; Ag 2 - C 24; AgNP 2 - Ag 24; Ag 24 - C 0; Ag 24 - C 2; Ag 24 - C 24; AgNP 2 - AgNPs 24; AgNP 2 - C 0; AgNP 2 - C 2; AgNP 2 - C 24; AgNPs 24 - C 0; AgNPs 24 - C 2; AgNPs 24 - C 24

CMP	42.246	3.5276e-08	7.4525	6.5325e-08	Ag 2 - Ag 24; Ag 2 - AgNPs 24; C 0 - Ag 2; C 2 - Ag 2; C 24 - Ag 2; AgNP 2 - Ag 24; AgNPs 24 - Ag 24; C 0 - Ag 24; C 2 - Ag 24; C 24 - Ag 24; AgNP 2 - AgNPs 24; C 0 - AgNP 2; C 2 - AgNP 2; C 24 - AgNP 2; C 0 - AgNPs 24; C 2 - AgNPs 24; C 24 - AgNPs 24
Serine	38.797	6.1489e-08	7.2112	1.098e-07	Ag 2 - Ag 24; Ag 2 - AgNPs 24; C 0 - Ag 2; C 2 - Ag 2; C 24 - Ag 2; AgNP 2 - Ag 24; C 0 - Ag 24; C 2 - Ag 24; C 24 - Ag 24; AgNP 2 - AgNPs 24; C 0 - AgNP 2; C 2 - AgNP 2; C 24 - AgNP 2; C 0 - AgNPs 24; C 2 - AgNPs 24; C 24 - AgNPs 24; C 0 - C 2
Guanine	32.899	1.7853e-07	6.7483	3.0782e-07	Ag 2 - Ag 24; Ag 2 - AgNPs 24; Ag 2 - C 0; Ag 2 - C 2; Ag 2 - C 24; AgNP 2 - Ag 24; Ag 24 - C 0; AgNP 2 - AgNPs 24; AgNP 2 - C 0; AgNP 2 - C 2; AgNP 2 - C 24; AgNPs 24 - C 0; C 2 - C 0; C 24 - C 0
Glutamic acid	31.64	2.2928e-07	6.6396	3.8213e-07	Ag 2 - Ag 24; Ag 2 - AgNP 2; Ag 2 - C 0; Ag 2 - C 2; Ag 2 - C 24; AgNPs 24 - Ag 24; Ag 24 - C 0; Ag 24 - C 2; Ag 24 - C 24; AgNPs 24 - AgNP 2; AgNP 2 - C 0; AgNP 2 - C 2; AgNP 2 - C 24; AgNPs 24 - C 0; AgNPs 24 - C 2; AgNPs 24 - C 24
Glucose/ Galactose	25.861	8.236e-07	6.0843	1.3284e-06	C 0 - Ag 2; C 2 - Ag 2; C 24 - Ag 2; C 0 - Ag 24; C 2 - Ag 24; C 24 - Ag 24; C 0 - AgNP 2; C 2 - AgNP 2; C 24 - AgNP 2; C 0 - AgNPs 24; C 2 - AgNPs 24; C 24 - AgNPs 24
Proline	22.515	1.9529e-06	5.7093	3.0515e-06	Ag 2 - Ag 24; Ag 2 - AgNP 2; C 0 - Ag 2; Ag 2 - C 2; Ag 2 - C 24; C 0 - Ag 24; Ag 24 - C 24; C 0 - AgNP 2; AgNP 2 - C 24; C 0 - AgNPs 24; AgNPs 24 - C 24; C 0 - C 2; C 0 - C 24; C 2 - C 24
Alanine	22.303	2.07e-06	5.684	3.1364e-06	Ag 2 - Ag 24; Ag 2 - AgNP 2; Ag 2 - AgNPs 24; C 0 - Ag 2; Ag 2 - C 24; AgNP 2 - Ag 24; C 0 - Ag 24; C 2 - Ag 24; C 24 - Ag 24; AgNP 2 - AgNPs 24; C 0 - AgNP 2; C 0 - AgNPs 24; C 2 - AgNPs 24; C 24 - AgNPs 24; C 0 - C 2; C 0 - C 24
Hypoxanthine	21.647	2.4885e-06	5.6041	3.6596e-06	Ag 2 - Ag 24; Ag 2 - AgNPs 24; Ag 2 - C 0; Ag 2 - C 2; Ag 2 - C 24; AgNP 2 - Ag 24; Ag 24 - AgNPs 24; Ag 24 - C 0; Ag 24 - C 24; AgNP 2 - AgNPs 24; AgNP 2 - C 0; AgNP 2 - C 2; AgNP 2 - C 24; AgNPs 24 - C 24; C 2 - C 0; C 2 - C 24
Ascorbic acid	19.7	4.428e-06	5.3538	6.3258e-06	Ag 2 - Ag 24; Ag 2 - AgNPs 24; Ag 2 - C 0; Ag 2 - C 2; Ag 2 - C 24; AgNP 2 - Ag 24; Ag 24 - C 2; AgNP 2 - AgNPs 24; AgNP 2 - C 0; AgNP 2 - C 2; AgNP 2 - C 24; C 0 - AgNPs 24; C 0 - C 2; C 0 - C 24
Malic acid	18.605	6.2583e-06	5.2035	8.6921e-06	Ag 2 - Ag 24; Ag 2 - AgNP 2; Ag 2 - AgNPs 24; Ag 2 - C 0; Ag 2 - C 2; Ag 2 - C 24; AgNP 2 - Ag 24; AgNPs 24 - Ag 24; C 0 - Ag 24; C 2 - Ag 24; C 24 - Ag 24; AgNP 2 - AgNPs 24; AgNP 2 - C 0; AgNP 2 - C 2; C 24 - AgNPs 24; C 24 - C 0; C 24 - C 2
Aspartic acid	12.973	5.1744e-05	4.2861	6.9924e-05	Ag 24 - Ag 2; Ag 2 - AgNP 2; AgNPs 24 - Ag 2; C 0 - Ag 2; Ag 24 - AgNP 2; Ag 24 - C 2; Ag 24 - C 24; AgNPs 24 - AgNP 2; C 0 - AgNP 2; C 2 - AgNP 2; C 24 - AgNP 2; AgNPs 24 - C 2; AgNPs 24 - C 24; C 0 - C 2; C 0 - C 24
Heptadecanoic acid	10.702	0.00015087	3.8214	0.00019851	Ag 24 - Ag 2; AgNPs 24 - Ag 2; Ag 24 - AgNP 2; Ag 24 - C 0; Ag 24 - C 2; Ag 24 - C 24; AgNPs 24 - AgNP 2; AgNPs 24 - C 0; AgNPs 24 - C 2; C 24 - C 0
Linolenic acid	9.7206	0.00025312	3.5967	0.00032452	Ag 24 - Ag 2; AgNPs 24 - Ag 2; Ag 24 - AgNP 2; Ag 24 - C 0; Ag 24 - C 2; Ag 24 - C 24; AgNPs 24 - AgNP 2; AgNP 2 - C 2; AgNPs 24 - C 0; AgNPs 24 - C 2; AgNPs 24 - C 24
Adenosine	9.0574	0.00036732	3.435	0.00045916	Ag 2 - Ag 24; C 0 - Ag 2; C 24 - Ag 2; AgNP 2 - Ag 24; C 0 - Ag 24; C 2 - Ag 24; C 24 - Ag 24; C 0 - AgNP 2; C 24 - AgNP 2; C 0 - AgNPs 24; C 24 - AgNPs 24; C 0 - C 2; C 24 - C 2
Thymine	8.4444	0.00052771	3.2776	0.00064355	Ag 2 - C 0; C 24 - Ag 2; C 24 - Ag 24; AgNP 2 - C 0; C 24 - AgNP 2; C 24 - AgNPs 24; C 2 - C 0; C 24 - C 0; C 24 - C 2

Uracil	8.0413	0.00067669	3.1696	0.00080559	Ag 2 - Ag 24; Ag 2 - AgNPs 24; Ag 2 - C 0; C 24 - Ag 24; AgNP 2 - C 0; C 24 - AgNP 2; C 24 - AgNPs 24; C 2 - C 0; C 24 - C 0; C 24 - C 2
Myristic acid	6.1261	0.0025207	2.5985	0.0029311	Ag 2 - AgNP 2; Ag 2 - C 0; Ag 2 - C 2; Ag 24 - AgNP 2; Ag 24 - C 0; Ag 24 - C 2; AgNPs 24 - AgNP 2; C 24 - AgNP 2; AgNPs 24 - C 0; C 24 - C 0; C 24 - C 2
Linoleic acid	5.0221	0.0060882	2.2155	0.0069184	Ag 24 - Ag 2; AgNPs 24 - Ag 2; C 24 - Ag 2; Ag 24 - C 0; C 24 - AgNP 2; AgNPs 24 - C 0; C 24 - C 0; C 24 - C 2
Palmitic acid	4.7251	0.0078663	2.1042	0.0087403	C 24 - Ag 2; Ag 24 - C 0; C 24 - AgNP 2; AgNPs 24 - C 0; C 24 - C 0; C 24 - C 2

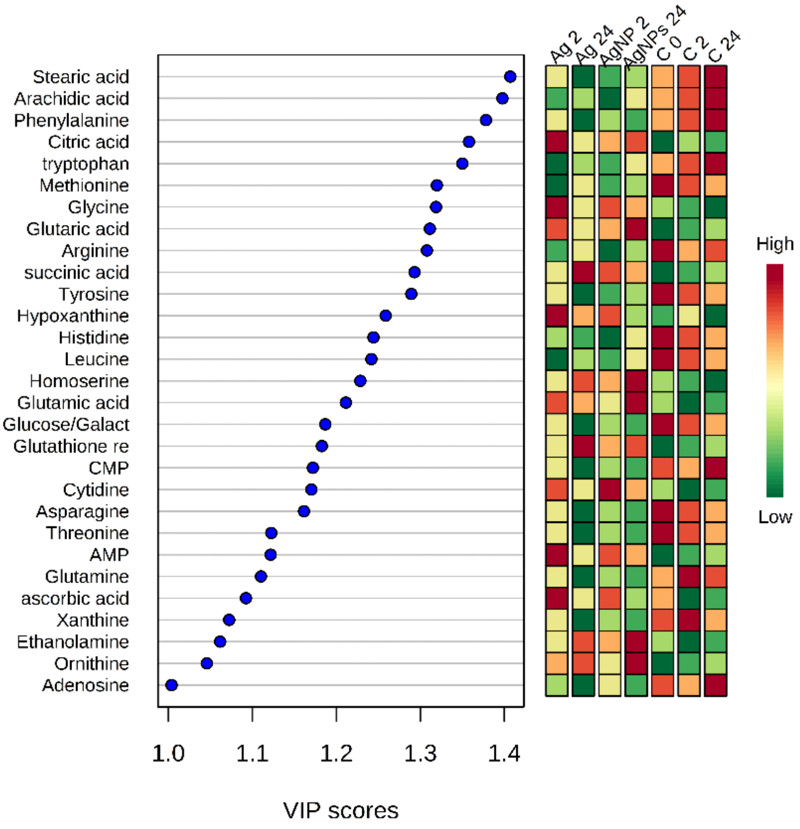

Figure S1. Important features identified by PLS-DA. The coloured boxes on the right indicate the relative concentrations of the corresponding metabolite in each group under study. VIP scores from PLS-DA analysis of discriminating metabolites between unexposed controls, 40.7 μgL^{-1} $AgNO_3$ and 1 mgL^{-1} AgNPs groups for 2h and 24h. Treatments: Ag2: 2h exposure to 40.7 μg$L^{-1}$$AgNO_3$; Ag24: 24h-exposure to 40.7 μgL^{-1} $AgNO_3$; AgNPs2: 2h exposure to 1 mgL^{-1}AgNPs; AgNPs24: 24h exposure to 1 mgL^{-1}AgNPs; C0: unexposed controls at time 0 (beginning of the experiment); C2: unexposed controls at time 2h; C24: unexposed controls at time 24h. Data were normalized with respect to unexposed control at time 0 (C0). The plot was generated by MetaboAnalyst 4.0 (https://www.metaboanalyst.ca/)[1]

2. Different groups of metabolites identified by cluster analysis

Different groups of metabolites were identified by cluster analysis (Fig. 4C). The first one represents the metabolites that were depleted under the Ag-treatments at 2 and 24h in comparison with unexposed controls and included 17 metabolites: adenosine, arachidic acid, arginine, asparagine, CMP, glucose/galactose, glutamine, histidine, leucine, methionine, phenylalanine, serine, stearic acid, threonine, tryptophan, tyrosine and xanthine. The second cluster corresponded to metabolites which accumulated during the Ag-treatments, which was more pronounced at 24h than at 2h exposure and included 10 metabolites: adenine, citric acid, citrulline, ethanolamine, glutamic acid, glutaric acid, glutathione reduced, homoserine, ornithine and succinic acid. The third cluster included, aspartic acid, heptadecanoic acid, linolenic acid and lysine, which significantly accumulated only during 24h exposure to AgNPs and dissolved Ag. The forth cluster included AMP, ascorbic acid, cytidine, glycine, guanine, hypoxanthine and malic acid which accumulated only during 2h exposure to AgNPs and dissolved Ag. More complex pattern was observed in the fifth cluster including thymine, uracil, myristic, linoleic and palmitic acids.

3. AgNPs and dissolved Ag induced changes in abundance of nucleotide/side metabolites

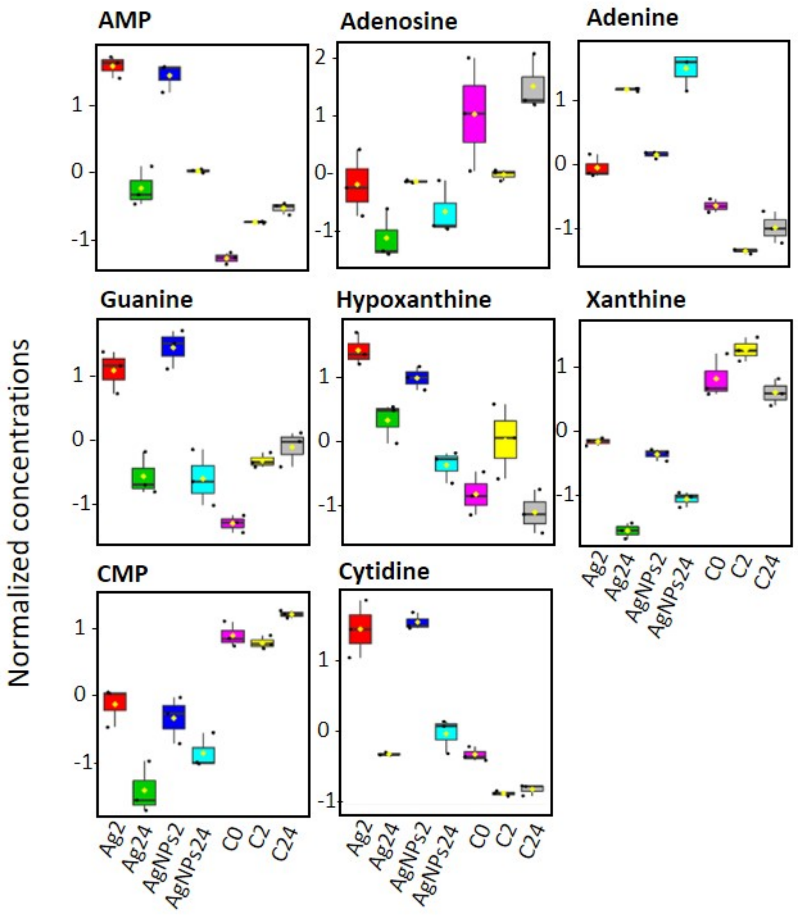

Figure S2. Box plots of relative abundance of nucleotide/side metabolites in *P. malhamensis* treated with 1mgL^{-1} AgNPs and 40.7 μgL^{-1}$AgNO_3$ for 2 and 24h and untreated controls. Treatments: Ag2: 2h exposure to 40.7 μgL^{-1}$AgNO_3$; Ag24: 24h-exposure to 40.7 μgL^{-1} $AgNO_3$; AgNPs2, 2h exposure to 1 mgL^{-1}AgNPs; AgNPs24; 24h exposure to 1 mgL^{-1}AgNPs; C0: unexposed controls at time 0 (beginning of the experiment); C2: unexposed controls at time 2h; C24: unexposed controls at time 24h. Data were normalized with respect to unexposed control at time 0 (C0).

4. AgNPs and dissolved Ag induced changes in abundance of fatty acids metabolites

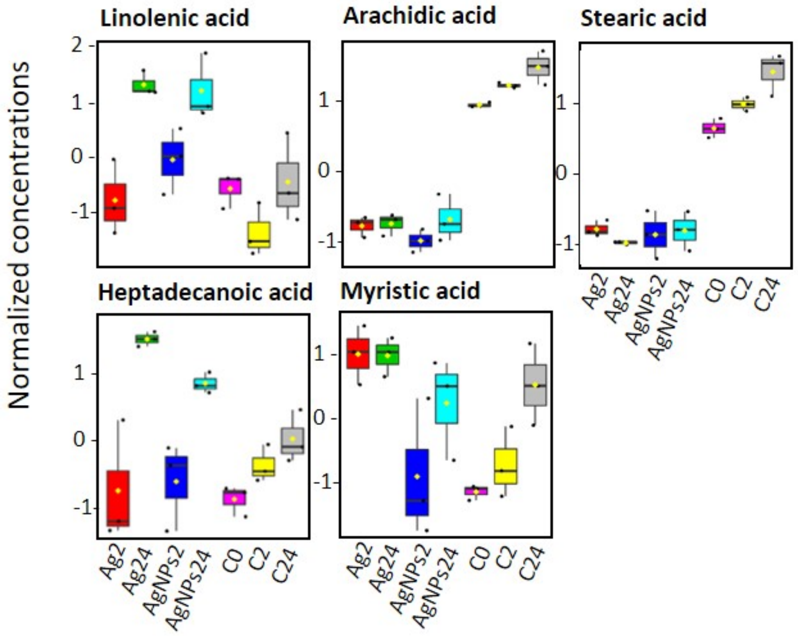

Figure S3. Box plots of relative abundance of fatty acids metabolites in *P. malhamensis* treated with 1mgL^{-1} AgNPs and 40.7 µgL^{-1}AgNO$_3$ for 2 and 24h and untreated controls untreated controls at time 0, 2 and 24h. Treatments: Ag2: 2h exposure to 40.7 µgL^{-1}AgNO$_3$; Ag24: 24h-exposure to 40.7 µgL^{-1} AgNO$_3$; AgNPs2: 2h exposure to 1 mgL^{-1}AgNPs; AgNPs24: 24h exposure to 1 mgL^{-1}AgNPs; C0: unexposed controls at time 0 (beginning of the experiment); C2: unexposed controls at time 2h; C24: unexposed controls at time 24h. Data were normalized with respect to unexposed control at time 0 (C0).

5. AgNPs and dissolved Ag induced changes in abundance of carboxylic acids metabolites

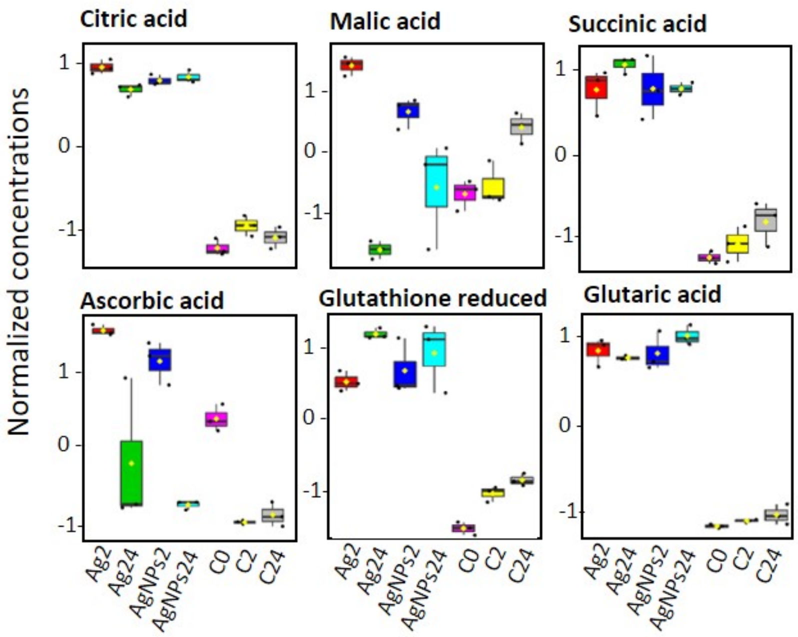

Figure S4. Box plots of relative abundance of carboxylic acids and antioxidants in *P. malhamensis* treated with 1mgL^{-1} AgNPs and 40.7 µgL^{-1}$AgNO_3$ for 2 and 24h and untreated controls at time 0, 2 and 24h. Treatments: Ag2: 2h exposure to 40.7 µgL^{-1}$AgNO_3$; Ag24: 24h-exposure to 40.7 µgL^{-1} $AgNO_3$; AgNPs2: 2h exposure to 1 mgL^{-1}AgNPs; AgNPs24: 24h exposure to 1 mgL^{-1}AgNPs; C0: unexposed controls at time 0 (beginning of the experiment); C2: unexposed controls at time 2h; C24: unexposed controls at time 24h. Data were normalized with respect to unexposed control at time 0 (C0).

6. Metabolic perturbations induced by 2h-exposure to AgNPs and dissolved Ag

Table S2. Important features identified by ANOVA and Fisher's post-hoc analysis in *P. malhamensis* exposed to 1 mgL^{-1} AgNPs (AgNP 2) and 40.7 μgL^{-1} $AgNO_3$ (Ag 2) and control at 2h exposure. Data were normalized with respect to unexposed control at time 2h (C2).

Metabolite	f.value	p.value	-LOG10(p)	FDR	Fisher's LSD
Homoserine	3169.8	8.4536e-10	9.073	4.2268e-08	AgNP 2 - Ag 2; Ag 2 - C 2; AgNP 2 - C 2
Tryptophan	1969.1	3.5204e-09	8.4534	8.801e-08	AgNP 2 - Ag 2; C 2 - Ag 2; C 2 - AgNP 2
Phenylalanine	1036.5	2.404e-08	7.6191	3.8943e-07	AgNP 2 - Ag 2; C 2 - Ag 2; C 2 - AgNP 2
Threonine	950.42	3.1154e-08	7.5065	3.8943e-07	Ag 2 - AgNP 2; C 2 - Ag 2; C 2 - AgNP 2
Tyrosine	518.32	1.9057e-07	6.72	1.9057e-06	C 2 - Ag 2; C 2 - AgNP 2
Citric acid	455.27	2.8054e-07	6.552	2.3378e-06	Ag 2 - C 2; AgNP 2 - C 2
Arginine	422.27	3.5104e-07	6.4546	2.5074e-06	Ag 2 - AgNP 2; C 2 - Ag 2; C 2 - AgNP 2
Ornithine	286.12	1.1172e-06	5.9519	6.9827e-06	Ag 2 - C 2; AgNP 2 - C 2
AMP	270.14	1.325e-06	5.8778	7.3609e-06	Ag 2 - C 2; AgNP 2 - C 2
Asparagine	247.61	1.7153e-06	5.7656	8.5767e-06	C 2 - Ag 2; C 2 - AgNP 2
Glutaric acid	175.52	4.7456e-06	5.3237	2.1571e-05	Ag 2 - C 2; AgNP 2 - C 2
Stearic acid	157.47	6.5339e-06	5.1848	2.5451e-05	C 2 - Ag 2; C 2 - AgNP 2
Ascorbic acid	156.79	6.6174e-06	5.1793	2.5451e-05	Ag 2 - AgNP 2; Ag 2 - C 2; AgNP 2 - C 2
Arachidic acid	145.91	8.1775e-06	5.0874	2.8123e-05	C 2 - Ag 2; C 2 - AgNP 2
Leucine	143.61	8.5684e-06	5.0671	2.8123e-05	AgNP 2 - Ag 2; C 2 - Ag 2; C 2 - AgNP 2
Cytidine	139.11	9.4081e-06	5.0265	2.8123e-05	Ag 2 - C 2; AgNP 2 - C 2
Xanthine	138.34	9.5617e-06	5.0195	2.8123e-05	C 2 - Ag 2; C 2 - AgNP 2
Glutamine	120.25	1.4423e-05	4.8409	4.0064e-05	Ag 2 - AgNP 2; C 2 - Ag 2; C 2 - AgNP 2
Methionine	103.98	2.205e-05	4.6566	5.8026e-05	C 2 - Ag 2; C 2 - AgNP 2
Glycine	97.457	2.6633e-05	4.5746	6.6583e-05	Ag 2 - C 2; AgNP 2 - C 2
Adenine	88.468	3.5282e-05	4.4524	8.4005e-05	Ag 2 - C 2; AgNP 2 - C 2
Guanine	84.821	3.9863e-05	4.3994	9.0598e-05	AgNP 2 - Ag 2; Ag 2 - C 2; AgNP 2 - C 2
Glucose	64.064	8.9516e-05	4.0481	0.00018735	C 2 - Ag 2; C 2 - AgNP 2
Citrulline	63.962	8.9926e-05	4.0461	0.00018735	AgNP 2 - Ag 2; Ag 2 - C 2; AgNP 2 - C 2
Succinic acid	45.5	0.00023666	3.6259	0.00047332	Ag 2 - C 2; AgNP 2 - C 2
Glutathione reduced	37.266	0.00041356	3.3835	0.00078105	Ag 2 - C 2; AgNP 2 - C 2
Ethanolamine	37.003	0.00042177	3.3749	0.00078105	Ag 2 - C 2; AgNP 2 - C 2
Histidine	34.985	0.00049262	3.3075	0.00087968	C 2 - Ag 2; C 2 - AgNP 2
Glutamic acid	31.655	0.00064873	3.1879	0.0011185	Ag 2 - AgNP 2; Ag 2 - C 2; AgNP 2 - C 2
Malic acid	28.414	0.00087092	3.06	0.0014515	Ag 2 - AgNP 2; Ag 2 - C 2; AgNP 2 - C 2
CMP	22.573	0.0016144	2.792	0.0026039	C 2 - Ag 2; C 2 - AgNP 2
Isoleucine	19.329	0.0024254	2.6152	0.0037896	C 2 - Ag 2; C 2 - AgNP 2
Serine	13.492	0.0060197	2.2204	0.0091208	C 2 - Ag 2; C 2 - AgNP 2
Hypoxanthine	8.1801	0.019321	1.714	0.028413	Ag 2 - C 2; AgNP 2 - C 2
Myristic acid	7.1776	0.025611	1.5916	0.036587	Ag 2 - AgNP 2; Ag 2 - C 2

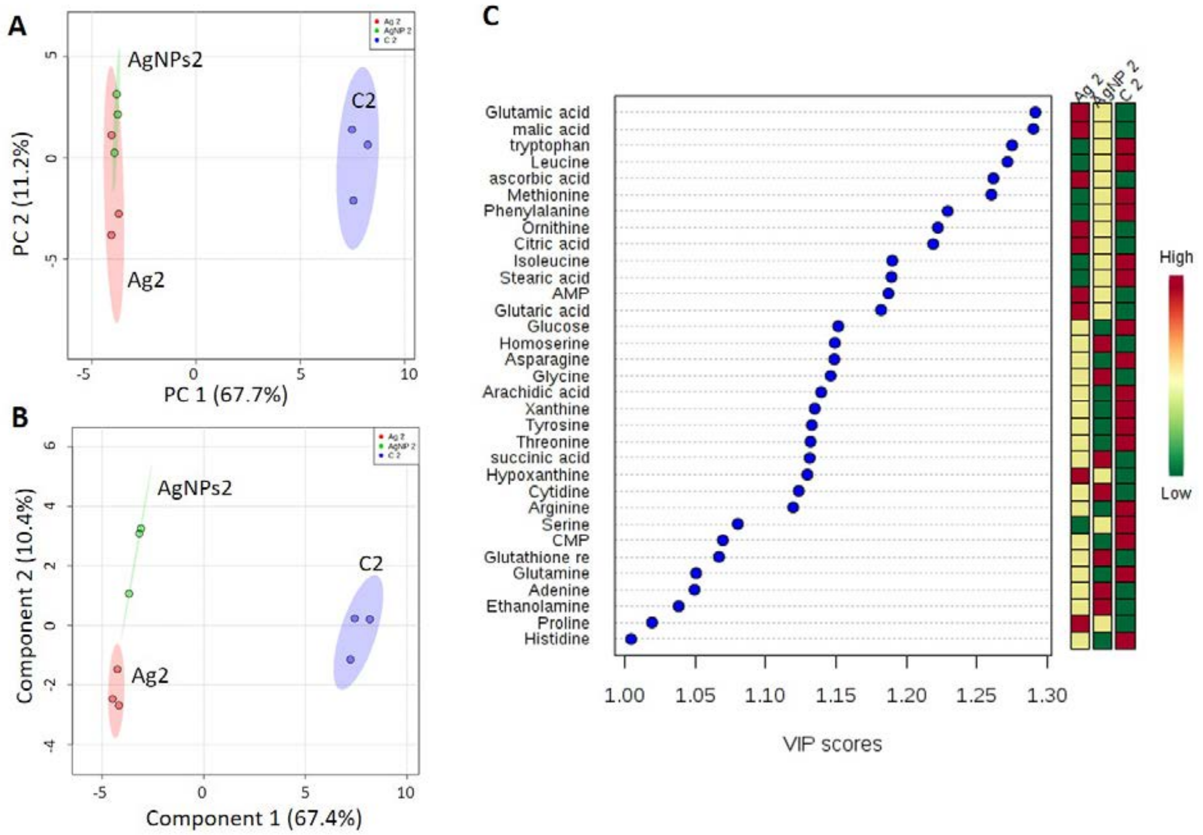

Figure S5. (A) Principal component analysis (PCA) and (B) partial least-squares discriminate analysis (PLS-DA) score plots of metabolic profiles in *P. malhamensis* treated with 1mgL^{-1} AgNPs and 40.7 µgL^{-1} $AgNO_3$ for 2h and untreated controls. Data were normalized by using Probabilistic Quotient Normalization by untreated control group at time 2h, log transformed and autoscaled. (C) Important features identified by PLS-DA. The coloured boxes on the right indicate the relative concentrations of the corresponding metabolite in each group under study. VIP scores from PLS-DA analysis of alga discriminating metabolites between unexposed control, 40.7 µgL^{-1} $AgNO_3$; and 1 mgL^{-1} AgNPs groups for 2h. Data were normalized with respect to unexposed control at time 2h (C2). The plots were generated by MetaboAnalyst 4.0 (https://www.metaboanalyst.ca/)[1]

7. Metabolic perturbations induced by 24h-exposure to AgNPs and dissolved Ag

Table S3. Important features identified by One-way ANOVA and Fisher's post-hoc analysis in *P. malhamensis* exposed to 1 mgL^{-1} AgNPs (AgNP 24) and 40.7 μgL^{-1} $AgNO_3$ (Ag 24) and control at 2h exposure. Data were normalized with respect to unexposed control at time 24h (C2).

Metabolite	f.value	p.value	-LOG(p)	FDR	Fisher's LSD
Homoserine	3351.6	7.1524e-10	9.1455	3.5762e-08	Ag 24 - C 24; AgNPs 24 - C 24
Arginine	489.35	2.2623e-07	6.6455	4.4063e-06	C 24 - Ag 24; C 24 - AgNPs 24
Lysine	458.58	2.7455e-07	6.5614	4.4063e-06	Ag 24 - AgNPs 24; Ag 24 - C 24; AgNPs 24 - C 24
Asparagine	421.68	3.525e-07	6.4528	4.4063e-06	C 24 - Ag 24; C 24 - AgNPs 24
Glutaric acid	357.68	5.7542e-07	6.24	5.2466e-06	AgNPs 24 - Ag 24; Ag 24 - C 24; AgNPs 24 - C 24
Phenylalanine	347.03	6.2959e-07	6.2009	5.2466e-06	C 24 - Ag 24; C 24 - AgNPs 24
Ornithine	272.4	1.2927e-06	5.8885	8.1895e-06	Ag 24 - C 24; AgNPs 24 - C 24
Histidine	271.15	1.3103e-06	5.8826	8.1895e-06	C 24 - Ag 24; C 24 - AgNPs 24
Citric acid	238.47	1.9177e-06	5.7172	9.6542e-06	Ag 24 - C 24; AgNPs 24 - C 24
Threonine	237.92	1.9308e-06	5.7143	9.6542e-06	C 24 - Ag 24; C 24 - AgNPs 24
Stearic acid	173.56	4.906e-06	5.3093	2.23e-05	C 24 - Ag 24; C 24 - AgNPs 24
Citrulline	153.96	6.9827e-06	5.156	2.9095e-05	Ag 24 - C 24; AgNPs 24 - C 24
Adenine	136.91	9.8593e-06	5.0062	3.792e-05	Ag 24 - C 24; AgNPs 24 - C 24
Xanthine	119.36	1.4737e-05	4.8316	5.0268e-05	AgNPs 24 - Ag 24; C 24 - Ag 24; C 24 - AgNPs 24
Glutamic acid	118.16	1.5182e-05	4.8187	5.0268e-05	AgNPs 24 - Ag 24; Ag 24 - C 24; AgNPs 24 - C 24
Succinic acid	113.5	1.7074e-05	4.7677	5.0268e-05	Ag 24 - C 24; AgNPs 24 - C 24
Arachidic acid	113.06	1.7269e-05	4.7627	5.0268e-05	C 24 - Ag 24; C 24 - AgNPs 24
Serine	111.27	1.8097e-05	4.7424	5.0268e-05	C 24 - Ag 24; C 24 - AgNPs 24
Ethanolamine	102.61	2.2919e-05	4.6398	6.0312e-05	AgNPs 24 - Ag 24; Ag 24 - C 24; AgNPs 24 - C 24
Glutamine	84.151	4.0789e-05	4.3895	9.7855e-05	AgNPs 24 - Ag 24; C 24 - Ag 24; C 24 - AgNPs 24
Tyrosine	83.931	4.1099e-05	4.3862	9.7855e-05	C 24 - Ag 24; C 24 - AgNPs 24
CMP	79.3	4.8436e-05	4.3148	0.00011008	AgNPs 24 - Ag 24; C 24 - Ag 24; C 24 - AgNPs 24
Adenosine	67.391	7.7412e-05	4.1112	0.00016829	C 24 - Ag 24; C 24 - AgNPs 24
Tryptophan	53.745	0.00014777	3.8304	0.00030785	C 24 - Ag 24; C 24 - AgNPs 24
Leucine	51.553	0.0001663	3.7791	0.00033261	C 24 - Ag 24; C 24 - AgNPs 24
Glutathione reduced	44.171	0.00025724	3.5897	0.0004947	Ag 24 - C 24; AgNPs 24 - C 24
Cytidine	39.948	0.00034083	3.4675	0.00063118	AgNPs 24 - Ag 24; Ag 24 - C 24; AgNPs 24 - C 24
Aspartic acid	35.732	0.00046466	3.3329	0.00082976	Ag 24 - C 24; AgNPs 24 - C 24
Glycine	34.722	0.00050303	3.2984	0.00086728	Ag 24 - C 24; AgNPs 24 - C 24
Heptadecanoic acid	29.312	0.00080034	3.0967	0.0013339	Ag 24 - AgNPs 24; Ag 24 - C 24; AgNPs 24 - C 24
Alanine	20.506	0.0020789	2.6822	0.0033531	AgNPs 24 - Ag 24; C 24 - Ag 24; C 24 - AgNPs 24
Proline	18.881	0.0025772	2.5889	0.0040268	Ag 24 - C 24; AgNPs 24 - C 24
Thymine	18.017	0.0029083	2.5364	0.0044065	C 24 - Ag 24; C 24 - AgNPs 24
Hypoxanthine	17.472	0.0031467	2.5021	0.0046275	Ag 24 - AgNPs 24; Ag 24 - C 24; AgNPs 24 - C 24
AMP	16.182	0.0038253	2.4173	0.0054647	AgNPs 24 - Ag 24; Ag 24 - C 24; AgNPs 24 - C 24
Uracil	14.135	0.0053665	2.2703	0.0074534	C 24 - Ag 24; C 24 - AgNPs 24
Methionine	13.679	0.0058189	2.2352	0.0078633	C 24 - Ag 24; C 24 - AgNPs 24
Linolenic acid	12.171	0.0077322	2.1117	0.010174	Ag 24 - C 24; AgNPs 24 - C 24
Glucose	11.597	0.0086811	2.0614	0.01113	C 24 - Ag 24; C 24 - AgNPs 24
Isoleucine	11.328	0.0091797	2.0372	0.011475	Ag 24 - C 24; AgNPs 24 - C 24
Malic acid	8.5007	0.01775	1.7508	0.021646	C 24 - Ag 24

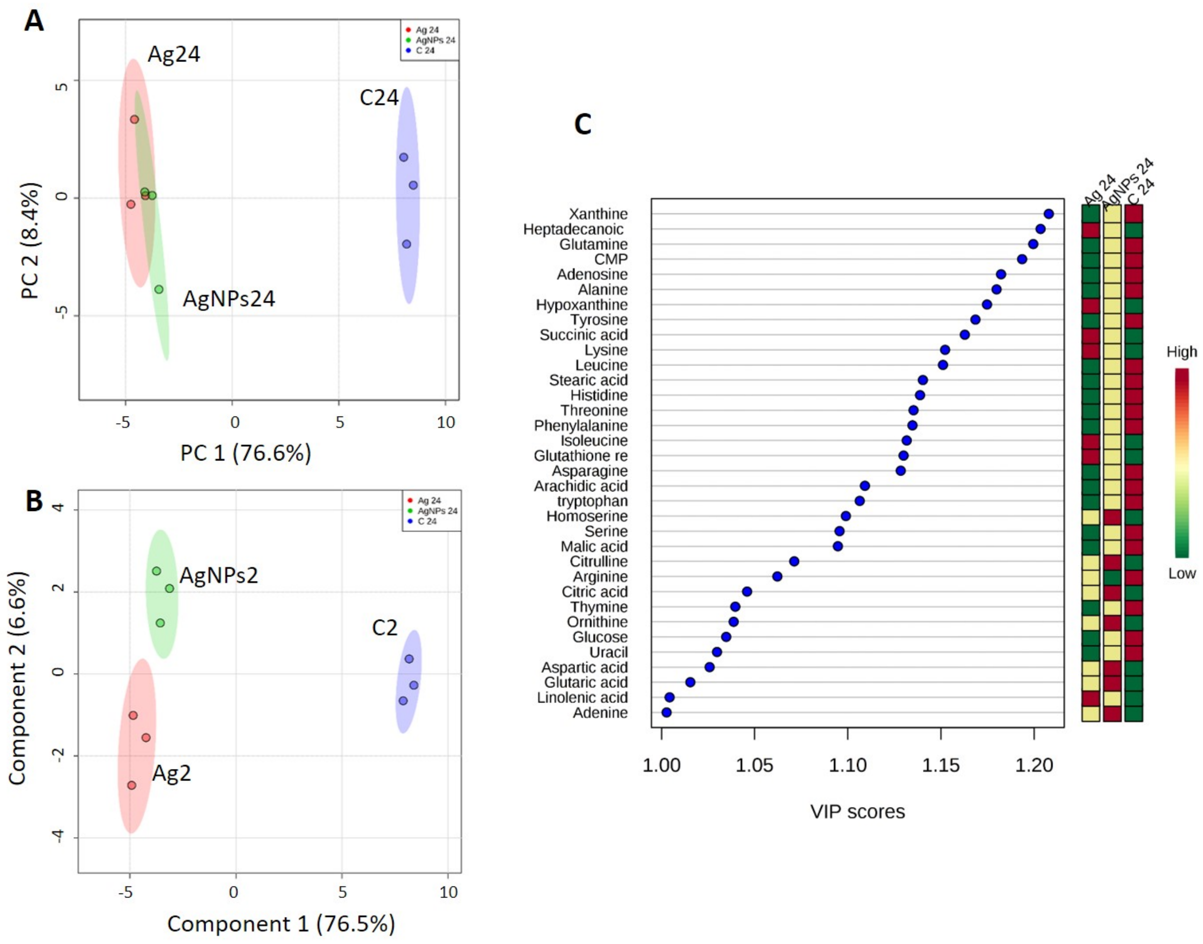

Figure S6. (A) Principal component analysis (PCA) and (B) partial least-squares discriminate analysis (PLS-DA) score plots of metabolic profiles in *P. malhamensis* treated with 1mgL^{-1} AgNPs and 40.7 µgL^{-1} $AgNO_3$ for 24h and untreated controls. Data were normalized by using Probabilistic Quotient Normalization by untreated control group at time 24h, log transformed and autoscaled. (C) Important features identified by PLS-DA. The colored boxes on the right indicate the relative concentrations of the corresponding metabolite in each group under study. VIP scores from PLS-DA analysis showing the discriminating metabolites between unexposed control, 40.7 µgL^{-1} $AgNO_3$; and 1 mgL^{-1} AgNPs. The plot were generated by MetaboAnalyst 4.0 (https://www.metaboanalyst.ca/)[1]

8. Metabolites common and specific for the two durations of exposure

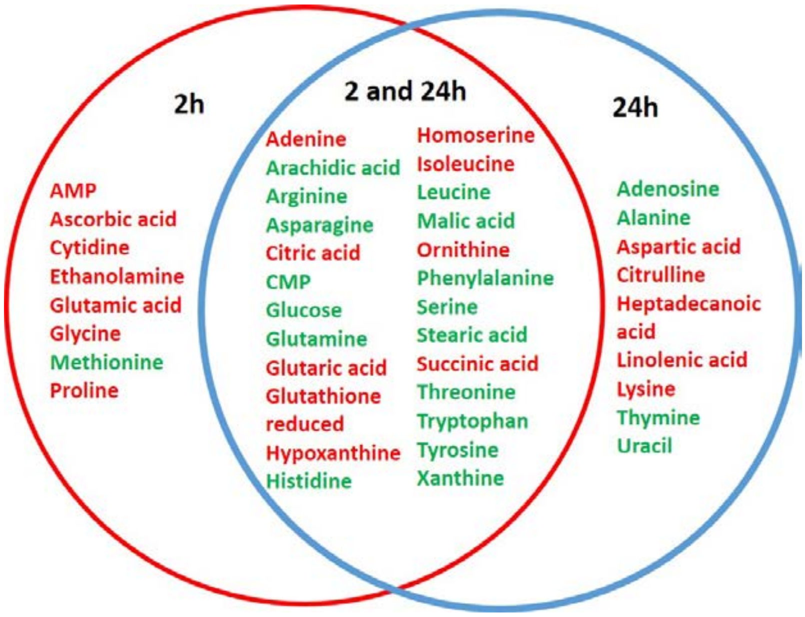

Figure S7. Metabolites that were significantly affected at 2 or 24h exposure and those that were common for two exposure durations identified by PLS-DA. Green and red colour represent the depleted and accumulated metabolites.

Twenty-five metabolites common for the two exposure times (Fig. S7): adenine, arachidic acid, arginine, asparagine, citric acid, CMP, glucose, glutamine, glutaric acid, glutathione reduced, hypoxanthine, histidine, homoserine, isoleucine, leucine, malic acid, ornithine, phenylalanine, serine, stearic acid, succinic acid, threonine, tryptophan, tyrosine and xanthine. AMP, ascorbic acid, cytidine, ethanolamine, glutamic acid, glycine, methionine and proline were specifically dysregulated at 2h exposure, while adenosine, aspartic acid, citrulline, heptadecanoic acid, linolenic acid, thymine and uracil at 24 h exposure. In addition, the ANOVA revealed that at 2h exposure 11 metabolites were significantly altered, relative to the control, by AgNPs and dissolved Ag: ascorbic acid, arginine, citrulline, glutamine, glutamic acid, guanine, homoserine, phenylalanine, threonine, tryptophan, and at 24h exposure: alanine, AMP, CMP, cytidine, glucose, glutamine, glutamic acid, glutaric acid, hypoxanthine, histidine, heptadecanoic acid and lysine.

9. Characteristics of stock suspension of AgNPs

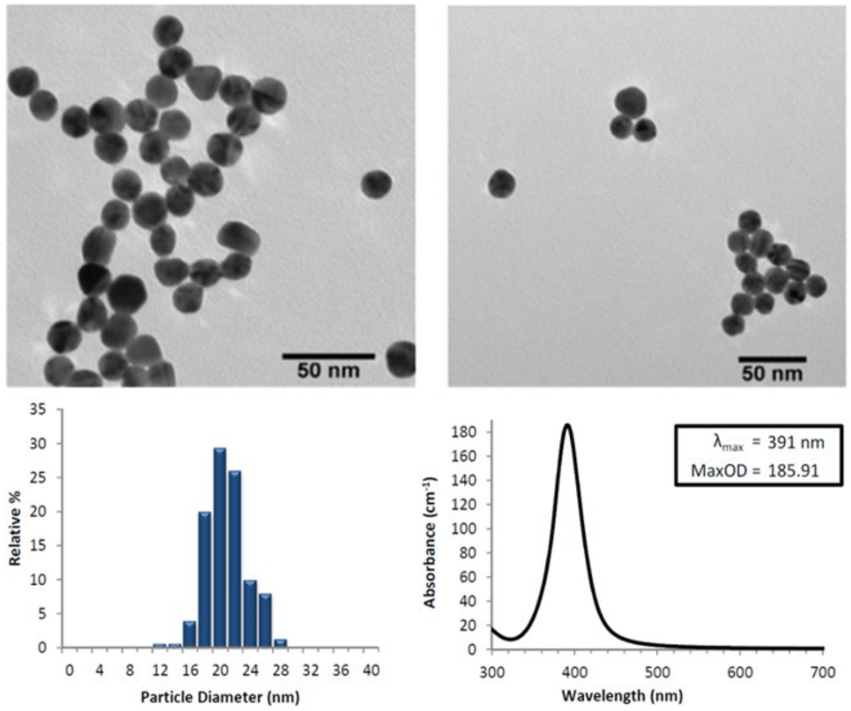

**Diameter (TEM):**	19.9 ± 2.8 nm	**Hydrodynamic Diameter:**	25 nm
**Coefficient of Variation:**	14.3 %	**Zeta Potential:**	-43 mV
**Surface Area (TEM):**	27.6 m^2/g	**pH of Solution:**	7.5
**Mass Concentration (Ag):**	1.04 mg/mL	**Silver Purity:**	99.99 %
**Particle Concentration:**	2.4E+13 particles/mL	**Particle Surface:**	Sodium Citrate
**Endotoxin Concentration:**	< 5 EU/mL	**Solvent:**	Aqueous 2mM Citrate

Figure S8. Characteristics of stock suspension of AgNPs according to the manufacturer.

10. Composition of the Waris-H medium

Table S4. Composition of the Waris-H medium (pH 7.0 ± 0.1) used in this study.

Compounds	Final Concentration	Compounds	Final Concentration
KNO_3	1.0 mM	$Na_2EDTA \cdot 2H_2O$	8.06 µM
$MgSO_4 \cdot 7H_2O$	81 µM	$FeSO_4 \cdot 7\ H_2O$	17.9 µM
$(NH_4)_2HPO_4$	0.15 mM	$MnCl_2 \cdot 4H_2O$	0.73 µM
$Ca(NO_3) \cdot 4\ H_2O$	0.42 mM	$ZnSO_4 \cdot 7H_2O$	73 nM
HEPES	1 mM	$CoCl_2 \cdot 6H_2O$	16.8 nM
H_3BO_3	18.43 µM	Vitamin B_{12}	0.15 nM
$Na_2SiO_3 \cdot 9\ H_2O$	0.5 mM	Thiamine · HCl	300 nM
Yeast extract	0.05%	Biotin	4.1 nM
Glucose	0.05%	Niacinamide	0.8 nM
Beef extract	0.05%	Peptone	0.4%

11. Determination of AgNPs dissolution in the exposure medium

Suspensions containing 0.5, 1 and 5 mgL^{-1} AgNPs were prepared by diluting the stock suspension in the Waris-H medium. The concentrations of the dissolved Ag in the AgNPs suspensions were determined at 2 and 24 h. To this end the aliquots of 6 mL of the suspensions were centrifuged at 165 000*g* for 4 h (Beckman Coulter, optimal L-100 × P ultracentrifuge) according to Beer et al. [2]. 1mL of the supernatant was collected, acidified with ultrapure HNO_3 to a final concentration of 1% (v/v). Ag concentrations in the supernatant were measured by inductively coupled plasma mass spectrometry (ICP-MS). The percentage of the dissolved Ag, calculated as a ratio of Ag concentration present in the supernatant to the total Ag concentration in the suspensions, is given in Table S2.

Table S5. Percentage of dissolved silver in AgNPs suspensions: mean and standard deviation (SD).

	Dissolved Ag (%)			
AgNPs	2h		24h	
mg L^{-1}	Mean	SD	Mean	SD
0.5	3.6	0.5	4.8	0.6
1	3.4	0.7	4.7	0.5
5	0.2	0.1	0.4	0.2

12. Silver cellular burden of *P. malhamensis* exposed to AgNPs for 2 and 24h

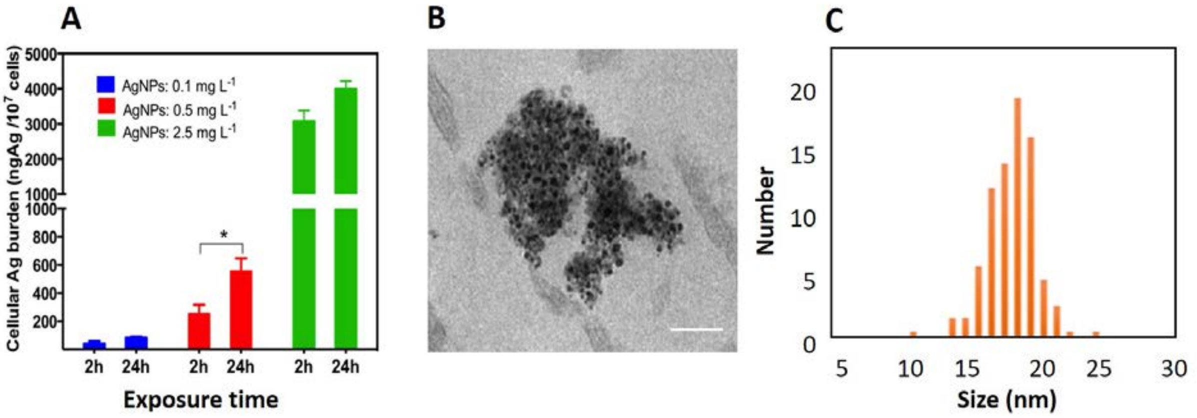

Figure S9. (A) Silver cellular burden at increasing AgNPs concentrations at 2 and 24h-exposure; (B) TEM image showing aggregates of AgNPs in the food vacuoles of *P. malhamensis*; (C) Size distribution of the aggregate obtained from the TEM image, present at Fig. S9B for algae exposed to 1 mg L^{-1} AgNPs during 24h. Asterisk indicates a significant difference between treatments obtained by two-way analysis of variance (ANOVA) followed by a Sidak's multiple comparisons test ($p < 0.05$).

13. Effect of AgNPs and dissolved Ag on algal growth

The algal cells at the mid-exponential phase were harvested by gently centrifugation and re-suspended in the modified Waris-H medium enriched with the AgNPs concentration ranging from 0.1 to 10 mgL^{-1} for 144 h. Cell numbers were followed and used to calculate the percentage of growth inhibition at different AgNPs concentrations over this time period. The experiments were performed in triplicates. The concentration inducing the growth inhibition in 50% of the algal population, EC_{50} (144h) of AgNPs was 7.76 mg L^{-1}. Exposure to 1 mgL^{-1} AgNPs resulted in a growth inhibition of 15% of the cells. The growth inhibition was also controlled when cells were exposed to 40.7μgL^{-1} $AgNO_3$, a concentration corresponding to the dissolved Ag in the suspensions of 1 mg L^{-1} AgNPs. At this concentration below 2% of the cells were affected. Based on these results we expect no effect on the algae growth under the selected exposure conditions of 2 and 24h duration and 1mgL^{-1} AgNPs and 40.7μgL^{-1} $AgNO_3$.

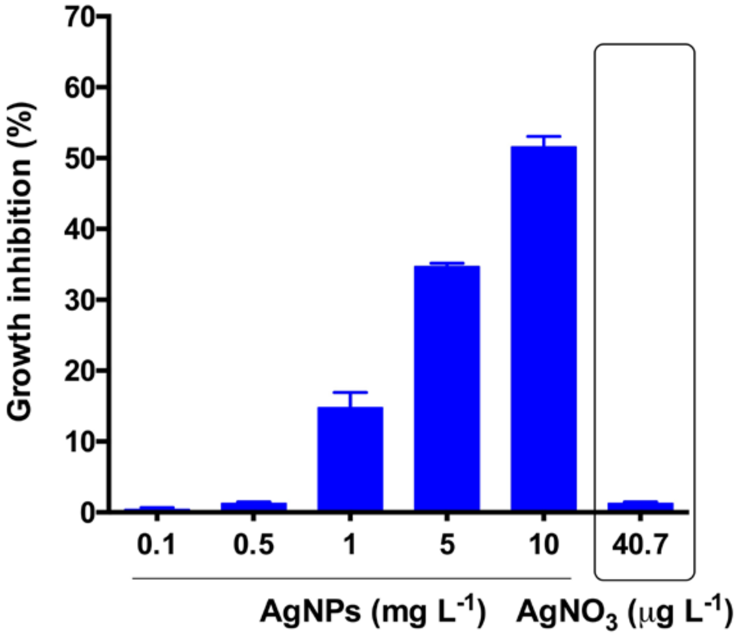

Figure S10. Growth inhibition of *P. malhamensis* exposed to AgNPs and $AgNO_3$ corresponding to the dissolved Ag in the 1 mg L^{-1} suspension of AgNPs or 40.7 μgL^{-1} $AgNO_3$. Error bars represent on standard deviation of three independent bioassays.

14. Determination of the cellular ROS upon AgNPs and dissolved Ag exposure

The generation of the cellular ROS was examined at 2h and 24h with the cell density of $1x10^6$ cells mL^{-1} using the fluorescent probe CellROX® green (Life Technologies Europe B.V., Zug, Switzerland), as previously detailed [3,4]. Briefly, 5µM CellROX green were employed to 500 µL aliquots of each test replicate separately for 30 minutes in the dark with no intermediate washing steps. Unexposed algae were used as negative control, while algae exposed to 5 mM H_2O_2 for 30 min were used as positive control. Measurements were performed with a BD Accuri C6 flow cytometer equipped with a CSampler (BD Biosciences, San Jose, CA). 488-nm argon excitation laser and fluorescence detection channels with band pass emission filters at 530 ± 15 nm (FL1), 585 ± 20 nm (FL2) and a long pass emission filter for >670 nm (FL3) were used. Data acquisition and analysis were performed with the BD Accuri C6 Software 264.15. Percentage of cells with enhanced ROS in different treatments is present at Figs. S11 and S12A.

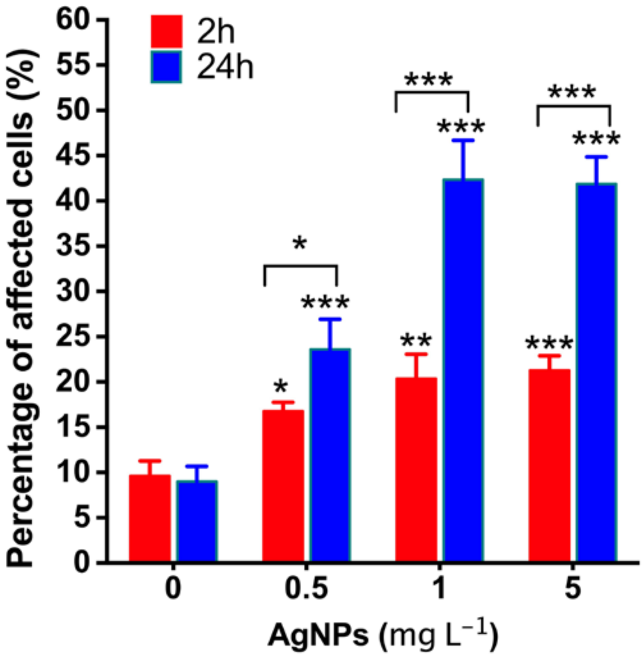

Figure S11. Percentage of cells affected by enhanced reactive oxygen species generation in the presence of AgNPs at 0.5, 1 and 5 mgL^{-1} at 2h and 24h-exposure. Asterisks indicate a significant difference between treatments obtained by two-way analysis of variance (ANOVA) followed by a Sidak's multiple comparisons test: *$p < 0.05$; *** $p < 0.01$; *** $p < 0.001$.

15. Determination of the lipid peroxidation upon AgNPs and dissolved Ag exposure

Cellular lipid peroxidation induced by Ag-treatments was determined following malondialdehyde (MDA) production MDA-assay kit (Sigma-Aldrich. St Louis, USA). The kit is based on the reaction of MDA with thiobarbituric acid, giving a product with a characteristic UV-visible absorbance peak at 532 nm, which was measured by spectrophotometry according to the manufacturer's instruction. The absorbance intensity at 532 nm is directly related to the amount of MDA formed. The assay was performed with 96 well microplate reader with a total volume of 200 µL of sample (Synergy H1, Biotek®, USA). The data were treated with Gen5 Software. The results are present in Fig. S12B.

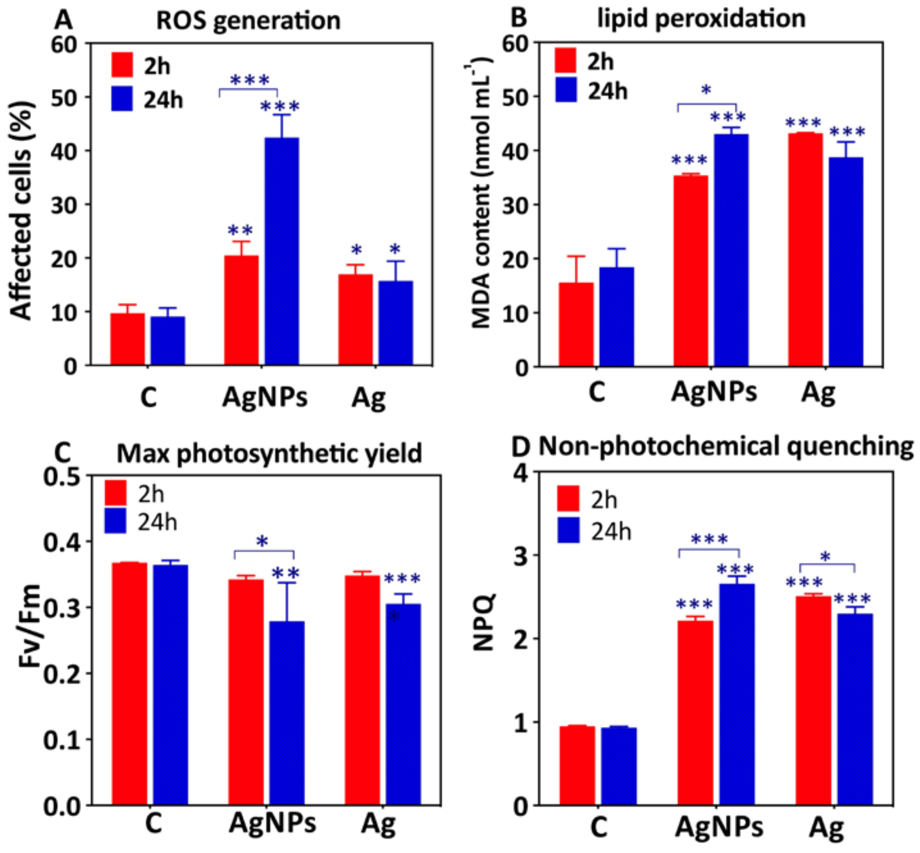

Figure S12. Effect of 1.0 mg L^{-1} AgNPs or 40.7 µg L^{-1} $AgNO_3$ exposure on physiology of *P. malhamensis*. (A): ROS generation, determined by CellRox® Green stain and FCM; (B) lipid peroxidation, assessed by MDA test; (C) Maximum quantum yield of photosystem II (Fv/Fm); and (D) non-photochemical quenching (NPQ). Asterisks indicate a significant difference between treatments obtained by two-way analysis of variance (ANOVA) followed by a Sidak's multiple comparisons test: *$p < 0.05$; *** $p < 0.01$; *** $p < 0.001$.

16. Determination of photosynthetic activity upon AgNPs and dissolved Ag exposure

Changes in the photosynthetic activities of *P. malhamensis* induced by Ag-treatments were measured using a Multi-PAM (Walz, Germany). Photosynthetic parameters including maximal efficiency of PSII (Fv/Fm) and non-photochemical quenching (NPQ), corresponding to the dissipation of the excess of energy as heat loss from PSII, were measured after 2h and 24h after dark acclimation for 20 min. The modulation of these parameters is a well-known indicator for stress induced by different biotic and abiotic factors. The results are present in Fig. S12C and D.

17. Metabolites and MS parameters for LC-MS targeted metabolomics

Table S6. List of metabolites and the MS parameters for LC-MS targeted metabolomics

Compound	Retention time (min)	Precursor ion (m/z)	Product ions				
			Quant ion (m/z)	Collision energy (V)	Qual ion (m/z)	Collision energy (V)	Fragmentor (V)
**Amino acids**							
Phenylalanine	2.95	166.1	120.1	13	103	29	80
Leucine	3.38	132.1	86.1	9	30.2	17	75
Tryptophan	3.41	205.1	188	8	146	20	80
Isoleucine	3.75	132.1	86.1	9	44.2	25	75
Methionine	4.22	150.1	104	9	56.1	17	75
Valine	4.95	118.1	72.1	9	55.1	25	70
Proline	4.96	116.1	70.1	17	43.2	37	75
Tyrosine	5.01	182.1	136.1	13	91.1	33	85
Cysteine	5.63	122	59.1	29	76	13	65
Alanine	6.61	90.1	44.2	9	45.3	40	40
Threonine	6.72	120.1	74.1	9	56.1	17	75
Homoserine	6.91	120.1	74.1	9	56.1	21	70
Glycine	7.00	76	30.3	12	-	-	35
Glutamine	7.23	147.1	84.1	17	130.1	9	80
Serine	7.26	106.1	88.1	8	42.2	24	67
Asparagine	7.31	133.1	87.1	5	74	17	75
Glutamic acid	7.68	148.1	84.1	17	130	5	75
Citrulline	7.89	176.1	159.1	9	70.1	25	80
Aspartic acid	8.38	134	88.1	9	74	13	70
Histidine	9.06	156.1	110.1	13	83.1	29	90
Arginine	9.54	175.1	70.1	24	60.1	12	100
Lysine	10.16	147.1	84.1	17	130.1	9	75
Ornithine	10.28	133.1	116	8	70	20	76
**Antioxidants**							
Glutathione reduced	1.22	308.1	179	12	162	16	91
Chlorogenic acid	6.19	353.1	191.1	16	-	-	102
Curcumin	6.33	367.1	217.1	8	149.1	16	112
Vanillic acid	6.60	167	152.1	12	108	20	82
2-hydroxycinnamic acid	7.37	163	119.1	12	117.1	28	81
L-Dehydroascorbic acid	8.00	173	158.1	12	-	-	174
4-(Trifuoromethyl)cinnamic acid	8.26	215	171.1	12	151.1	20	87
a-Tocopherol	11.00	431.4	165.1	24	69.1	40	142
**Organic Acids/Phenolics**							
glycolic acid	2.04	75	47	8	72.9	8	46
malic acid	2.07	133	114.9	8	71	16	76

Citric acid	2.17	191	110.8	12	86.9	16	82
lactic acid	2.23	89.1	43.1	4	-	-	66
succinic acid	2.31	117	72.9	12	98.9	8	66
Pyruvic acid	2.36	87	43.1	4	-	-	66
Gallic acid	2.49	169	125.1	12	79	24	92
Glutaric acid	2.62	131	86.9	12	112.9	8	71
fumaric acid	2.67	115	70.9	4	-	-	56
ascorbic acid	2.67	175	114.9	12	-	-	87
Caffeic acid	4.58	179	135.1	16	-	-	94
p-coumaric acid	4.87	163	119.1	16	93.1	36	87
ferulic acid	5.09	193.1	134.1	16	178.1	12	87
benzoic acid	5.21	121	77.1	12	-	-	77
Salicyllic acid	5.96	137	93	20	65.1	36	82
**Amine**							
Ethanolamine	1.23	62.1	44.2	8	45.2	16	66
2.4-Diaminoanisole	1.25	139.1	124	16	79	32	71
4.4'-Diaminodiphenylmethane	1.27	199.1	106	28	77	40	127
4.4'-Oxydianiline	1.28	201.1	108	24	80	40	117
m-Phenylenediamine	1.30	109.1	92	16	65	28	76
Aniline	1.57	94.1	77	20	51.1	36	40
o-Anisidine	1.59	124.1	109	16	80	36	61
o-Toluidine	1.67	108.1	91	20	65	32	91
4-Chloroaniline	2.17	128	93	20	75	40	86
2.6-Dimethylaniline	2.18	122.1	105	16	77	32	86
2-Methyl-5-nitroaniline	2.38	153.1	107	20	89	40	71
Diphenylamine	3.41	170.1	93	28	65.1	36	132
**Sugar and Sugar Alcohol**							
Ribose	1.18	149	89	4	-	-	76
L-fucose	1.35	163.1	89	0	59.1	12	76
Arabinose	1.43	149	89	4	-	-	76
Xylose	1.43	149	89	4	-	-	76
Ribitol	1.61	151.1	89	8	71.1	16	97
Xylitol	1.61	151.1	89	12	-	-	97
Fructose	1.72	179.1	89	4	-	-	71
Mannose	1.93	179.1	89	16	-	-	71
Galactose	2.19	179.1	89	16	-	-	71
Glucose	2.19	179.1	89	16	-	-	71
Sucrose	3.81	341.1	179	20	-	-	148
Maltose	4.26	341.1	161.1	4	-	-	123
Lactose	4.57	341.1	161.1	4	-	-	123
Trehalose	4.79	341.1	179	12	-	-	154
Raffinose	6.03	503.2	179	20	221	32	174
Galactinol	6.17	341.1	179	12	-	-	133

**Fatty Acids**							
Linolenic acid	4.33	323.2	277.1	4	45.1	40	87
myristic acid	4.64	273.2	227.2	4	45.1	8	56
Linoleic acid	4.91	325.2	279.1	4	45.1	28	87
Pentadecanoic acid	5.17	287.2	241.2	4	45.1	16	71
Palmitic acid	5.70	301.2	255.2	4	45.1	20	36
Heptadecanoic acid	6.14	315.3	269.2	4	45.2	28	76
Stearic acid	6.49	329.3	283.2	4	45.1	32	72
Arachidic acid	7.05	357.3	311.3	4	45.1	32	82
**Nucleobase/side/tide**							
Cytosine	1.94	112.1	95	20	40.1	20	84
CMP	2.76	324.1	112	16	95	40	84
Cytidine	2.90	244.1	112	12	95	40	84
Adenine	3.08	136.1	119	24	92	32	84
Guanine	3.34	152.1	135	20	110	24	84
Uracil	3.52	113	70	10	96	20	84
AMP	4.84	348.1	136	20	97	32	84
Hypoxanthine	5.28	137	110	24	55.1	36	148
Uridine	6.33	245.1	113	8	70	40	84
Xanthine	6.40	153	110	20	55.1	36	84
Adenosine	6.67	268.1	136	20	119	40	84
Thymine	6.71	127.1	110	16	54.1	28	84
Guanosine	6.91	284.1	152	12	135	40	84
Inosine	6.91	269.1	137	16	110	40	84
Thymidine	7.28	243.1	127	8	117	8	84

References

1 Xia, J. & Wishart, D. S. Web-based inference of biological patterns, functions and pathways from metabolomic data using MetaboAnalyst. *Nature Protocols* **6**, 743, doi:10.1038/nprot.2011.319 (2011).

2 Beer, C., Foldbjerg, R., Hayashi, Y., Sutherland, D. S. & Autrup, H. J. T. l. Toxicity of silver nanoparticles—nanoparticle or silver ion? *Toxicology letters* **208**, 286-292 (2012).

3 Cheloni, G. & Slaveykova, V. I. Photo-Oxidative Stress in Green Algae and Cyanobacteria. *Reactive Oxygen Species* **5** (2018).

4 Cheloni, G., Marti, E. & Slaveykova, V. I. Interactive effects of copper oxide nanoparticles and light to green alga Chlamydomonas reinhardtii. *Aquatic Toxicology* **170**, 120-128, doi:10.1016/j.aquatox.2015.11.018 (2016).
